# Identification of two early blood biomarkers *ACHE* and *CLEC12A* for improved risk stratification of critically ill COVID-19 patients

**DOI:** 10.1038/s41598-023-30158-1

**Published:** 2023-03-16

**Authors:** Simone Kattner, Jan Müller, Karolina Glanz, Mehdi Manoochehri, Caroline Sylvester, Yevhen Vainshtein, Marc Moritz Berger, Thorsten Brenner, Kai Sohn

**Affiliations:** 1 Department of Anesthesiology and Intensive Care Medicine, University Hospital Essen, University Duisburg-Essen, Essen, Germany; 2 Innovation Field In-Vitro Diagnostics, Fraunhofer Institute for Interfacial Engineering and Biotechnology IGB, Stuttgart, Germany; 3 Center for Integrative Bioinformatics Vienna (CIBIV), Max Perutz Labs, University of Vienna and Medical University of Vienna, Vienna BioCenter, Vienna, Austria; 4 Vienna BioCenter PhD Program, Doctoral School of the University of Vienna and Medical University of Vienna, Vienna, Austria

**Keywords:** Predictive markers, Transcriptomics

## Abstract

In order to identify biomarkers for earlier prediction of COVID-19 outcome, we collected blood samples from patients with fatal outcomes (non-survivors) and with positive clinical outcomes (survivors) at ICU admission and after seven days. COVID-19 survivors and non-survivors showed significantly different transcript levels for 93 genes in whole blood already at ICU admission as revealed by RNA-Seq. These differences became even more pronounced at day 7, resulting in 290 differentially expressed genes. Many identified genes play a role in the differentiation of hematopoietic cells. For validation, we designed an RT-qPCR assay for C-type lectin domain family 12 member A (*CLEC12A*) and acetylcholinesterase (*ACHE*), two transcripts that showed highest potential to discriminate between survivors and non-survivors at both time points. Using our combined RT-qPCR assay we examined 33 samples to accurately predict patient survival with an AUROC curve of 0.931 (95% CI = 0.814–1.000) already at ICU admission. *CLEC12A* and *ACHE* showed improved prediction of patient outcomes compared to standard clinical biomarkers including CRP and PCT in combination (AUROC = 0.403, 95% CI = 0.108–0.697) or SOFA score (AUROC = 0.701 95% CI = 0.451–0.951) at day 0. Therefore, analyzing *CLEC12A* and *ACHE* gene expression from blood may provide a promising approach for early risk stratification of severely ill COVID-19 patients.

## Introduction

In March 2020 the World Health Organization (WHO) declared a pandemic due to severe acute respiratory syndrome coronavirus 2 (SARS-CoV-2)^1^. By January 2022, SARS-CoV-2 infected about 349 million people and caused more than 5.6 million deaths^2^. The coronavirus disease 2019 (COVID-19) causes several respiratory and systemic symptoms with widely varying degrees of severity, ranging from mild common cold up to the development of severe hypoxemia with acute respiratory distress syndrome (ARDS) and multiple organ failure (MOF)^3^. Dramatic progressions of the disease are hallmarked by an overwhelming innate inflammatory response in both, the lungs as well as the bloodstream, which is mainly driven by immune cells such as neutrophils or monocytes/macrophages. These cells recognize a broad range of pathogen- and damage-associated molecular patterns (PAMPS, DAMPS) via cell-surface bound pattern recognition receptors (PRR), promoting multisystemic immune dysregulation with an uncontrolled cytokine release (such as interleukin (IL)-6, tumor necrosis factor (TNF)-α or interferons (IFN)). This cytokine storm leads to a clinical picture of viral sepsis and is characterized by local death of epithelial cells and immune thrombosis in the lungs of patients suffering from severe COVID-19. Accordingly, the extent of this cytokine storm is known to be associated with disease severity and is inversely correlated with patient’s outcome^4,5^. Therefore, immune-inflammatory biomarkers such as IL-6, C-reactive protein (CRP), or procalcitonin (PCT) have already been used for predicting the extent of lung involvement and clinical outcomes in patients with COVID-19^6–11^. For better adaptation of the host response to a disease-causing virus and to reduce collateral damage, the proinflammatory response is accompanied by an interferon (IFN)-mediated antiviral response^12^. However, these innate antiviral responses (including type I interferons (IFNα/β) or type III IFN (IFNλ) have been shown to be impaired in the early phase of COVID-19, contributing to a viral persistence with an overwhelming host-damaging proinflammatory response^12^. Consequently, patient’s immune balance is of crucial importance for further disease progression. Accordingly, in-depth knowledge of patient’s immune status might be helpful for risk stratification and outcome prediction in COVID-19. In this context, Next-Generation Sequencing (NGS)-based RNA-Seq analyses of patient-specific RNA signatures allow for detailed profiling of the host response on the transcriptional level during viral infections. It is therefore hypothesized, that NGS-based gene expression analyses might allow for reliable risk stratification, prognosis estimation, and treatment guidance in patients suffering from COVID-19^13–15^.

Therefore, the aims of the present study were (i) identification of host signatures on the transcriptomic level for risk assessment and prognostic potential in COVID-19 patients using comprehensive NGS-based RNA-Seq analyses of survivors and non-survivors at the ICU, (ii) to compare RNA-signatures with standard of care diagnostics including C-reactive protein (CRP), procalcitonin (PCT), and sequential organ failure assessment (SOFA) score as well as to (iii) establish an easy to implement RT-qPCR approach for identified prognostic transcripts.

## Results

### Differences of COVID-19 survivors compared to non-survivors on the transcriptomic level

**Figure 1:**
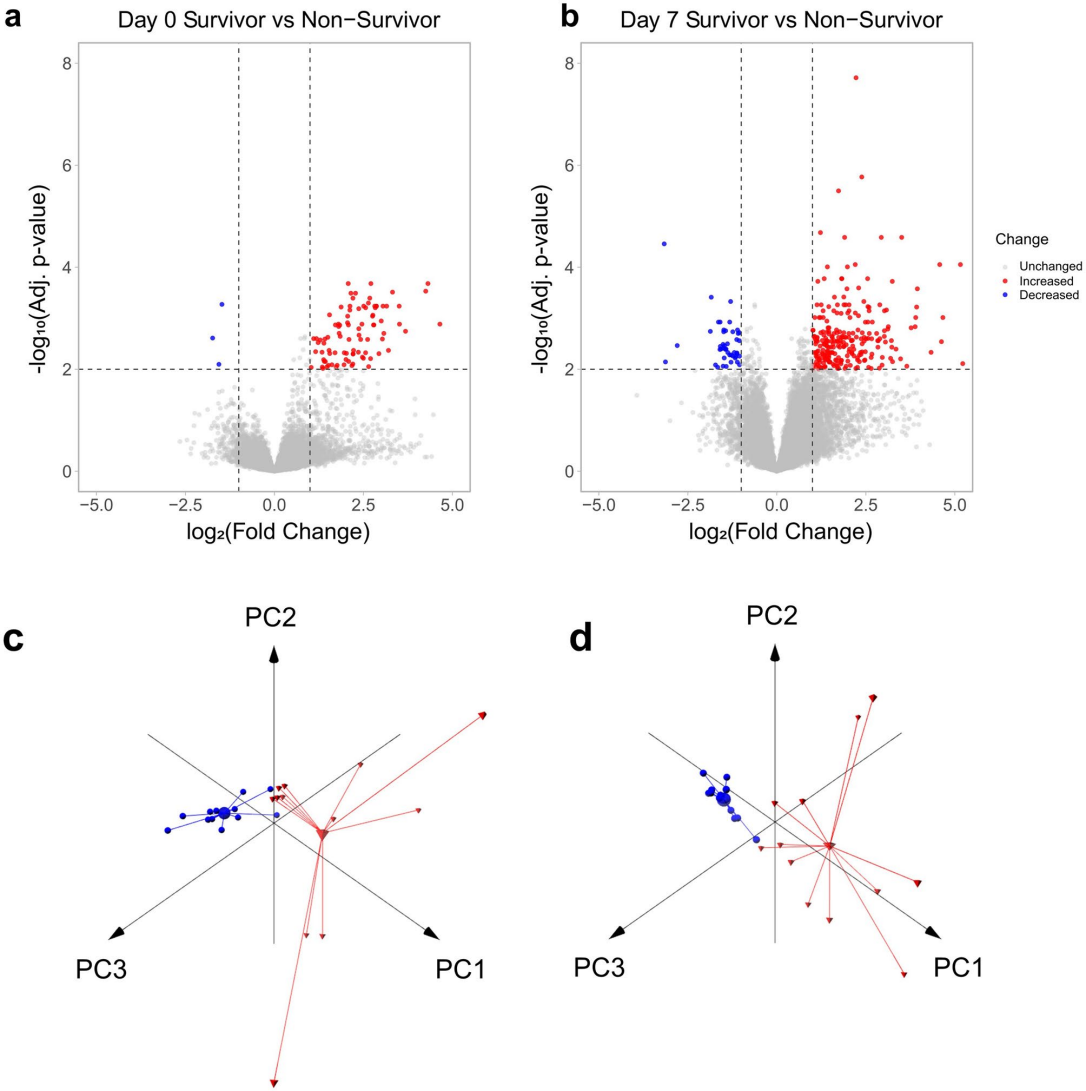
Differential expression analysis and principal component analysis of COVID-19 patients at day 0 and day 7 of ICU admission. (**a**) Volcano plot showing the transcriptomic differences of COVID-19 patients upon ICU admission (day 0, n = 24 (survivor n = 12, non-survivor n = 12)). (**b**) Volcano plot showing the transcriptomic differences of COVID-19 patients at day 7 in ICU (n = 24 (survivor n = 12, non-survivor n = 12)). Blue dots represent differentially expressed genes with a significantly increased expression level in survivors, while red dots represent genes with a significantly increased expression level in non-survivors (adjusted *p-*value ≤ 0.01 and a log_2_ fold change ≤  − 1 or ≥ 1). (**c**) Principal component analysis using the differentially expressed genes between COVID-19 patients with different outcomes at day 0 (% variance explained: PC1 = 72.44, PC2 = 7.69, PC3 = 4.15). (**d**) Principal component analysis using the differentially expressed genes between COVID-19 patients with different outcomes at day 7 (% variance explained: PC1 = 43.17, PC2 = 13.29, PC3 = 9.37). Blue spheres indicate survivors and red pyramids represent non-survivors. A centroid is shown for both conditions, connected to each data point by a line.

**Figure 2:**
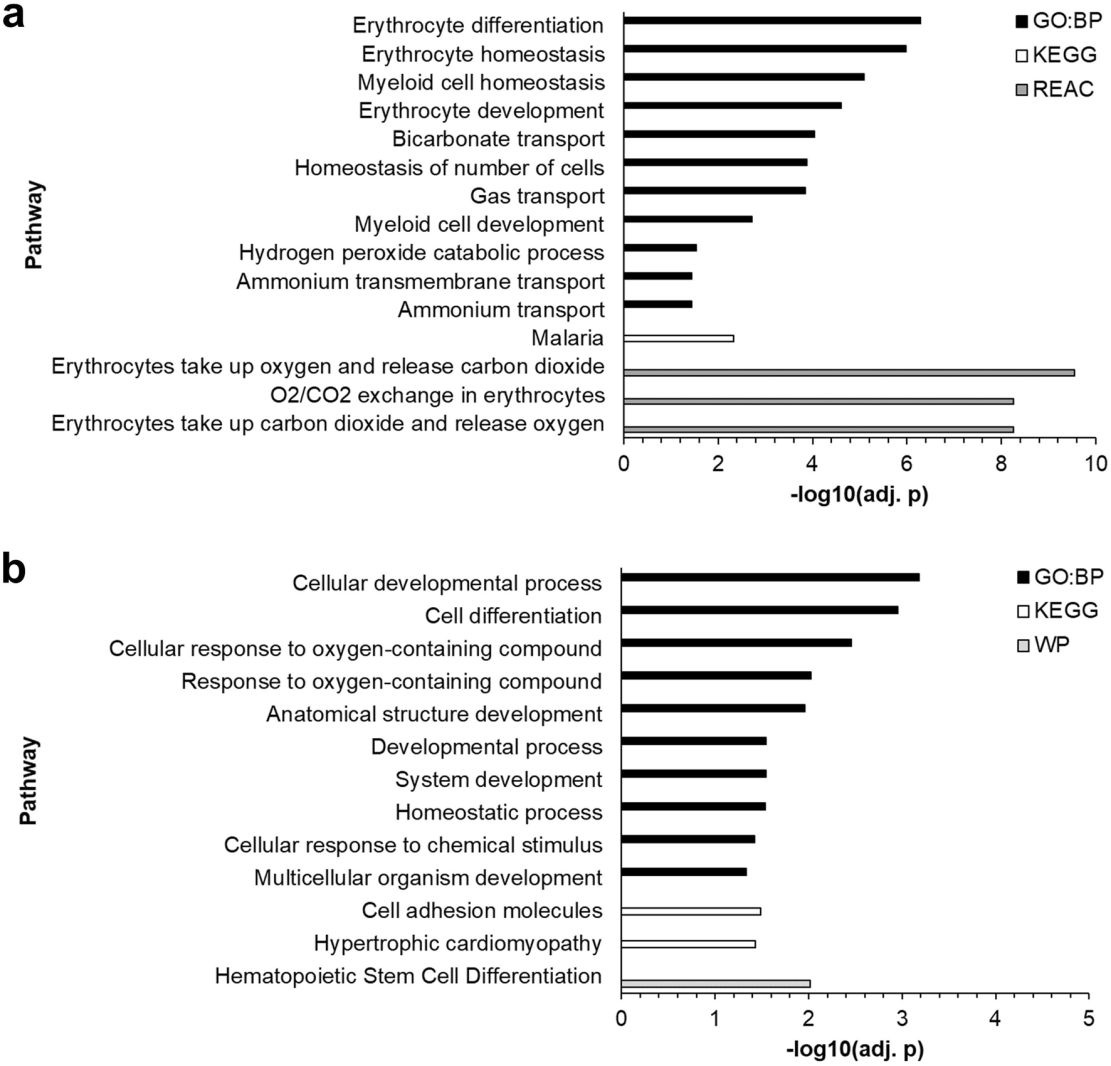
Pathway enrichment analysis for differentially expressed genes**.** (**a**) 93 differentially expressed genes at day 0 between COVID-19 survivors and non-survivors were evaluated for enrichment of pathways. (**b**) 290 differentially expressed genes at day 7 between COVID-19 survivors and non-survivors were evaluated for enrichment of pathways. GeneOntology (Biological Processes), KEGG, Reactome, and Wikipathways were used in this over-representation analysis for both DEG sets. Only pathways with an adjusted *p-*value ≤ 0.05 for enrichment are shown.

In an exploratory phase of this study, we performed RNA Next-Generation Sequencing (RNA-Seq) on intracellular mRNA from whole blood of patients to identify transcriptomic differences in the host-response of COVID-19 survivors and non-survivors (Supplementary Figure S1). Survivors and non-survivors were also tested for putative co-infections by analyzing microbial cell-free DNA (cfDNA) from blood plasma through NGS-based high-throughput sequencing. Recently, this approach proved to be the most sensitive and specific method for pathogen detection compared to standard microbiological culturing methods ^16,17^. However, co-infections occurred only sporadically, showing no significant difference between survivors and non-survivors. The vast majority of microbes detected are known contaminating species. At day 0, for only two patients of COVID-19 survivors, we detected a significant microbial signal, of which one microbe is not known to cause infections in humans (*Cupriavidus necator*). Only one patient of COVID-19 non-survivors was found to have a significant signal for *Human betaherpesvirus 5,* reflecting rather an incompetent immune response and the severity of the underlying disease than a co-infection (see Supplementary Data). On the contrary, RNA-Seq analysis for coding genes revealed significant differential expression for 93 genes at day 0 with at least a twofold increase or decrease and an adjusted *p-*value ≤ 0.01 when comparing survivors with non-survivors (Fig. 1a). At day 7, the number of differentially expressed genes (DEGs) increased to 290, indicating a more significant difference in host responses correlating with severity and outcome between the two cohorts at later time points (Fig. 1b). Principal component analysis (PCA) based on these DEGs for each time point showed a clear separation of patients with different clinical outcomes (Fig. 1c, d). The PCA provides a first indication that differences in the transcriptomes of both patient groups can be used for reliable classification and that these markers thus provide discriminatory power. In line with a higher number of DEGs at day 7, the separation of both groups was even more pronounced at day 7 with a higher intra-conditional variance for non-survivors compared to survivors (Fig. 1c, d). In order to identify biological processes involved in different clinical outcomes, functional enrichment analysis was performed using DEGs from both time points. At admission to the ICU, several significantly overrepresented pathways indicated changes in O_2_/CO_2_ exchange in erythrocytes and cellular differentiation of erythrocytes (adj. *p *= 5.6⋅10^–9^). All DEGs involved were more highly expressed in non-survivors, suggesting an adaptation to overcome an oxygen deficiency by increasing the gas exchange potential of erythrocytes and the production of additional erythrocytes (Fig. 2a). After seven days on the ICU, several signaling pathways indicated altered cellular differentiation, particularly of hematopoietic stem cells, including erythrocyte development genes that were more highly expressed in non-survivors (adj. *p *= 0.00096). Additionally, non-survivors showed a higher expression of genes involved in hypertrophic cardiomyopathy (Fig. 2b).

### Early biomarkers for the prediction of outcome in patients suffering from severe COVID-19

**Figure 3:**
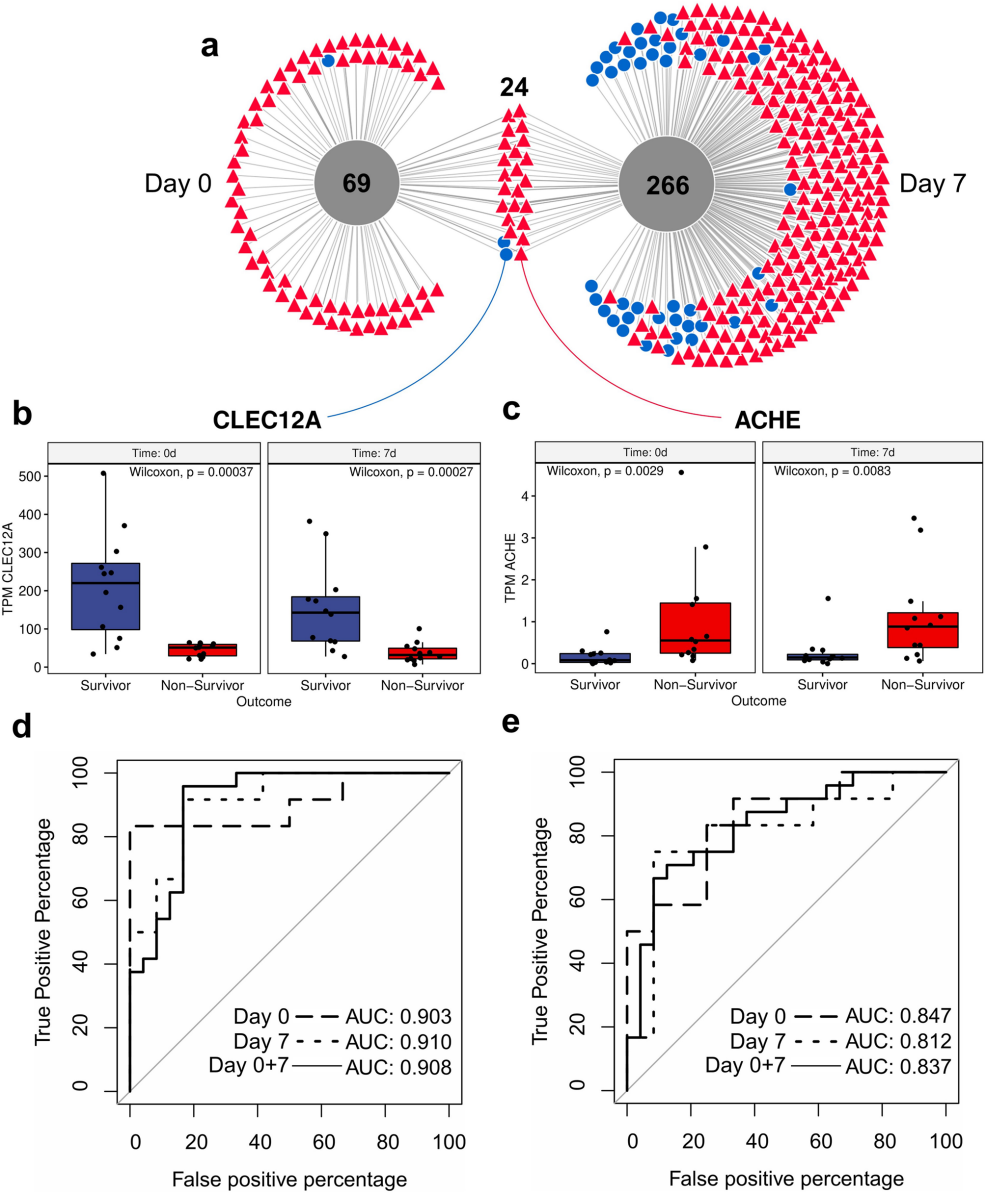
Identification of the putative biomarkers *CLEC12A* and *ACHE*. (**a**) Venn diagram comparing DEGs of COVID-19 patients at day 0 and day 7. 24 out of 359 DEGs were identified at both time points. Blue circles indicate genes with high expression in survivors and red triangles represent genes with high expression in non-survivors. (**b**) Boxplot showing the significantly decreased mean expression of *CLEC12A* in the blood transcriptome of non-survivors (red) separately for day 0 and day 7. (**c**) Boxplot showing the significantly increased mean expression of *ACHE* in the blood transcriptome of survivors (blue) separately for day 0 and day 7. Two-sided Wilcoxon test with n = 24 (survivor n = 12, non-survivor n = 12) was performed for both time points. Additionally, unequal variance t-tests were performed for both time points (p_ACHE,day0_ = 0.0019, p_ACHE,day7_ = 0.0063, p_CLEC12A,day0_ = 0.0002, p_CLEC12A,day7_ = 0.0001). (**d**) ROC curves for predicting the outcome of COVID-19 patients using *CLEC12A* at day 0, day 7, and for both time points combined. (**e**) ROC curves for predicting the outcome of COVID-19 patients using *ACHE* at day 0, day 7, and for both time points combined.

**Figure 4:**
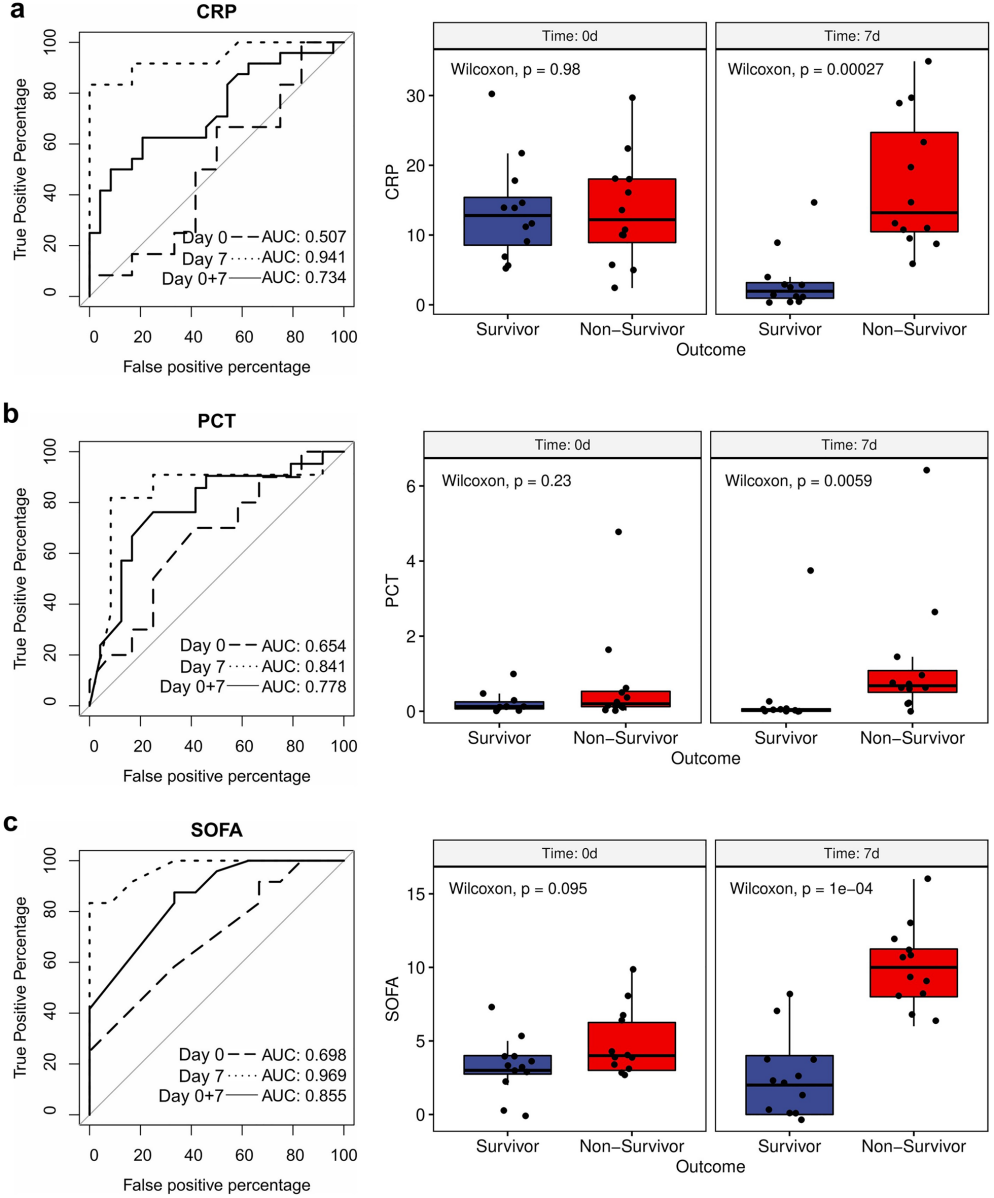
ROC curve analysis and boxplots for the evaluation of three standard of care markers for patients with different clinical outcomes. (**a**) Evaluation for C-reactive protein (CRP) for survivors and non-survivors at different sampling times. Values are shown in mg/dL. (**b**) Evaluation for procalcitonin (PCT) for survivors and non-survivors at different sampling times. Values are shown in ng/mL. (**c**) Evaluation for sequential organ failure assessment (SOFA) score for survivors and non-survivors at different sampling times. ROC curve analysis was performed for both time points separately (day 0 = long dashed line, day 7 = short dashed line) and combined (day 0 + 7 = solid line). Both sampling time points are shown separately in two boxplots. Differences between the mean values of the two outcome groups (survivors and non-survivors) were assessed with a two-sided Wilcoxon test separately for day 0 and day 7 with n = 24 (survivors n = 12, non-survivors n = 12). Additionally, unequal variance t-tests were performed (p_CRP,day0_ = 0.96, p_CRP,day7_ = 1.45⋅10^–5^, p_PCT,day0_ = 0.72, p_PCT,day7_ = 0.02, p_SOFA,day0_ = 0.09, p_SOFA,day7_ = 1.14⋅10^–6^).

As COVID-19 progression is unique to each patient admitted to the ICU, we aimed to identify early biomarkers that might discriminate survivors from non-survivors at various time points. Therefore, we considered DEGs that were differentially expressed in the same manner at both time points and with mean expression levels which did not differ significantly between day 0 and day 7 (Supplementary Figure S2). Of the 359 DEGs, 24 met these criteria and two were higher expressed in COVID-19 survivors, whereas 22 were higher expressed in non-survivors (|log_2_FC|≥ 1 and adjusted *p-*value ≤ 0.01, Fig. 3a-c, Supplementary Table S3). To determine the potential of DEGs to predict patients’ outcomes, we applied receiver operating characteristic (ROC) analyses (Fig. 3d, e). The area under the ROC curves (AUROC) using data from both time points for all 24 DEGs are summarized in Supplementary Table S3. The four most promising biomarker genes to predict clinical outcome in COVID-19 patients were C-type lectin domain family 12 member A (*CLEC12A*, AUROC = 0.908, 95% CI = 0.822–0.994), rhesus blood group CcEe antigen (*RHCE*, AUROC = 0.875, 95% CI = 0.774–0.976), C-type lectin domain family 12 member B (*CLEC12B*, AUROC = 0.863, 95% CI = 0.754–0.972), and acetylcholinesterase (*ACHE*, AUROC = 0.837, 95% CI = 0.722–0.951). *CLEC12A* and *CLEC12B* showed higher expression in survivors, whereas *RHCE* and *ACHE* were more highly expressed in non-survivors. Evaluation of the prognostic potential of COVID-19 severity biomarkers CRP, PCT and the SOFA score showed, that these readily available standard of care markers were not able to reliably predict patient outcome at day 0, with AUROC curve values comparable to random classification (CRP AUROC = 0.507 95% CI = 0.261–0.753, PCT AUROC = 0.654 95% CI = 0.416–0.892, SOFA AUROC = 0.698 95% CI = 0.492–0.904). Only on day seven, standard of care markers are strong predictors of patient outcome (CRP AUROC = 0.941 95% CI = 0.844–1.000, PCT AUROC = 0.841 95% CI = 0.647–1.000, SOFA AUROC = 0.969 95% CI = 0.914–1.000); Fig. 4).

### Establishment of an RT-qPCR test for CLEC12A and ACHE

**Figure 5:**
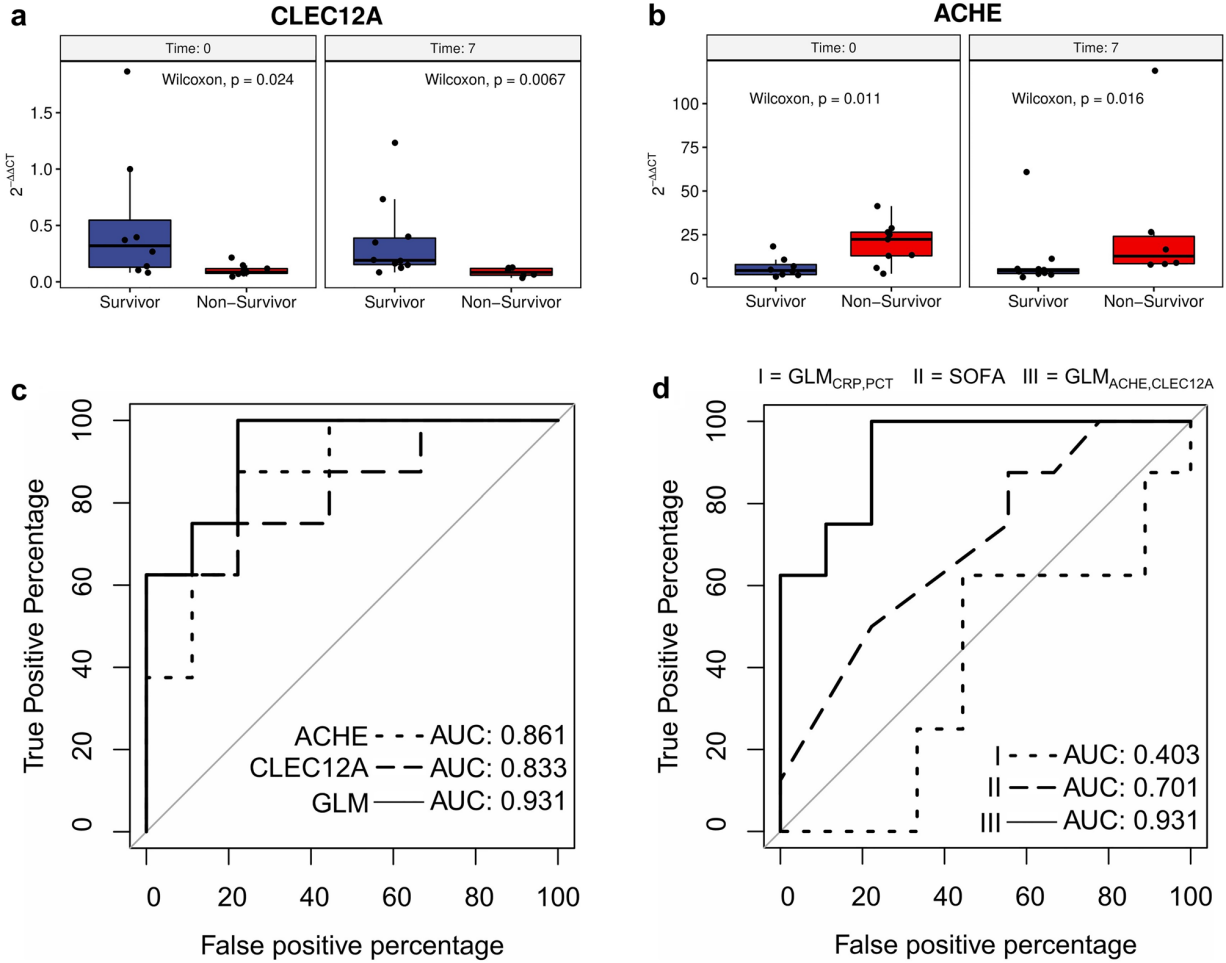
RT-qPCR validation of biomarkers *CLEC12A* and *ACHE*. (**a**) Boxplot showing significantly decreased mean abundance of *CLEC12A* transcripts in the blood of non-survivors in comparison to survivors (two-sided Wilcoxon test with a total n = 33 (survivors n = 18 (day 0 n = 8, day 7 n = 10), non-survivors n = 15 (day 0 n = 9, day 7 n = 6)); unequal variance t-test (p_CLEC12A_ = 0.017, p_ACHE_ = 0.007)). (**b**) Boxplot showing significantly increased mean abundance of ACHE transcripts in the blood of non-survivors in comparison to survivors (two-sided Wilcoxon test with a total n = 33 (see a); unequal variance t-test (p_CLEC12A_ = 0.001, p_ACHE_ = 0.005)). The RT-qPCR values of *CLEC12A* and *ACHE* were normalized to the two housekeeping genes *FPGS* and *PEX16* to calculate a normalized 2^-ΔΔCT^ value. (**c**) ROC curves for predicting the outcome of COVID-19 patients at day 0 using *ACHE*, *CLEC12A*, and a generalized linear model (GLM) that combines both biomarkers. (**d**) ROC curves for predicting the outcome of COVID-19 patients at day 0 using a GLM based on CRP and PCT (GLM_CRP,PCT_ = I), SOFA score (II), and a GLM of *ACHE* and *CLEC12A* (GLM_ACHE,CLEC12A_ = III).

For clinical validation and a potential translation from bench to bedside of the testing procedure, we developed a targeted RT-qPCR assay for *CLEC12A* and *ACHE*, as their classification performance (AUROC) in combination with signal difference at day 0 (FC) was the strongest. The RT-qPCR assay confirmed our RNA-Seq results, and differences between both clinical outcome groups were significant for *CLEC12A* and *ACHE* (*CLEC12A,* median log_2_FC = -1.89, *p *= 0.024 (day 0) and median log_2_FC = − 1.18, *p *= 0.007 (day 7); *ACHE,* median log_2_FC = 2.30, *p *= 0.011 (day 0) and median log_2_FC = 1.53, *p *= 0.016 (day 7), Fig. 5a, b). The expression of *CLEC12A* was shown to be significantly lower in independent healthy control samples than in COVID-19 survivors or non-survivors at day 0 and day 7 (healthy controls vs. COVID-19 survivors: p_day0_ = 0.00097, p_day7_ = 0.00028; healthy controls vs. COVID-19 non-survivors: p_day0_ = 0.005, p_day7_ = 0.039; Supplementary Figure S3b, d). The same applies to *ACHE*, except for COVID-19 survivors at day 0 (healthy controls vs. COVID-19 survivors: p_day0_ = 0.064, p_day7_ = 0.015; healthy controls vs. COVID-19 non-survivors: p_day0_ = 7.6⋅10^–5^, p_day7_ = 0.00088; Supplementary Figure S3a, c). Time-combined AUROC of *ACHE* and *CLEC12A* were comparable to RNA-Seq data (*ACHE*, AUROC = 0.848, 95% CI = 0.708–0.988; *CLEC12A*, AUROC = 0.874, 95% CI = 0.756–0.992; Supplementary Figure S4). Combining both biomarkers with a generalized linear model (GLM) as proposed by Mazzara et al*.* further improved the classification performance (AUROC = 0.963, 95% CI = 0.908–1.000; Fig. 5c)^18^. Comparison of *CLEC12A*, *ACHE*, and the combination thereof with CRP and PCT in combination showed a significantly better classification performance at day 0 (GLM_ACHE,CLEC12A_ AUROC = 0.931 95% CI = 0.814–1.000, GLM_CRP,PCT_ AUROC = 0.403 95% CI = 0.108–0.697; Fig. 5d**;** GLM_ACHE,CLEC12A_ vs. GLM_CRP,PCT_: *p-*value = 0.0017). Additionally, our newly identified biomarkers *CLEC12A* and *ACHE* demonstrated in combination a significantly larger AUROC curve than the SOFA score at day 0 (SOFA AUROC = 0.403 95% CI = 0.108–0.697; Fig. 5d; GLM_ACHE,CLEC12A_ vs. SOFA: *p-*value = 0.0469). Hence, the two-gene-signature *CLEC12A* and *ACHE* could provide an accurate tool for stratifying COVID-19 patients already upon ICU admission and at least one week earlier than CRP, PCT, and SOFA.

## Discussion

This study identified clear differences in blood cell transcriptomes from COVID-19 patients with fatal outcomes compared to COVID-19 patients who recovered at the ICU. Additionally, we revealed robust biomarkers for clinical stratification that are already discriminatory at ICU admission and thus account for differences in COVID-19 disease progressions. RNA-Seq data from an exploratory phase of this study with 24 patients revealed 93 differentially expressed genes at day 0 and 290 differentially expressed genes at day 7 between survivors and non-survivors, indicating a more pronounced separation of both groups as disease progressed. We performed a functional enrichment analysis for a better understanding of the underlying physiological differences that lead to the observed transcriptomic differences. We found that O_2_/CO_2_ exchange capacity in erythrocytes and the cellular differentiation to erythrocytes were mainly affected. These changes likely reflect acute respiratory distress syndrome (ARDS)-associated hypoxemia in non-survivors, promoting the development of megakaryoerythroid progenitor cells and proerythroblasts into erythrocytes and affecting hemoglobin levels in erythrocyte progenitor cells^19^. Seven days later, the transcriptomic differences between the different patient outcome groups mainly pointed towards cellular differentiation, especially of hematopoietic stem cells, which was still induced by hypoxemia. In the context of COVID-19, the relationship between different degrees of hypoxemia and mortality has already been demonstrated^20^. However, we found that the most promising classifiers for clinical outcome, *CLEC12A,* and *ACHE*, were not directly linked to hypoxia, but were involved in the regulation of the patient's immune response.

We demonstrated decreased expression of *CLEC12A* in COVID-19 non-survivors in the ICU. CLEC12A belongs to the dectin gene cluster and is a transmembrane cell surface receptor expressed mainly on myeloid cells that binds monosodium urate (MSU) crystals. MSU crystals are DAMPs formed after the release of soluble uric acid from dead cells. Whether there is a causal link between the recently reported low serum uric acid levels of COVID-19 patients, which appear to be strongly associated with disease severity and its progression to death, and the decreased expression of *CLEC12A* cannot be fully answered^21^. Still, such a physiological alteration could affect expression of *CLEC12A*. Alternatively, a reported reduction in *CLEC12A* expression upon inflammatory stimuli could also be a possible cause for the lower expression levels in COVID-19 patients with worse outcome^22,23^. CLEC12A acts anti-inflammatory by limiting the neutrophil recruitment to damaged tissue through inhibition of CXCL1 and CXCL10 production. In addition, IL-8 and reactive oxygen species (ROS) production in activated neutrophils is limited by inhibition of tyrosine-protein kinase SYK. Thus the NF-κB pathway is inhibited by the CLEC12A-recruited tyrosine phosphatases SHP-1 and SHP-2^24,25^. For example, critically ill patients have excessive levels of ROS, leading to tissue damage, thrombosis, and red blood cell dysfunction, which in turn contribute to the severity of COVID-19^26^. On the other hand, CLEC12A enhances the IFN response following viral infection, thereby enhancing the antiviral immune response, which is diminished and delayed in patients with COVID-19 and is only observed in patients developing critical illness^12,27^. A recent study found that the SARS-CoV-2 S protein can bind to the C-type lectin receptors (CLRs) DC-SIGN, L-SIGN, and CLEC10A. The binding promotes the expression of inflammatory cytokines in myeloid cells, which correlates with disease severity^28^. CLRs act in concert with Toll-like receptors (TLRs), thereby promoting inflammation and contributing to the pathological amplification of inflammatory responses^29^. These findings highlight the importance of CLRs and their effect on the immune response for COVID-19. By detecting cell death, CLEC12A constitutes an immune checkpoint that provides a negative feedback mechanism for immune regulation and tissue protection from an excessive inflammatory response^30^. Protection from an overwhelming immune response in COVID-19 is critical for patient survival and the regulation of inflammatory factors, like IL-8, ROS, or CXCL10 by CLEC12A might play an important functional role, besides its utility as a biomarker for outcome prediction in critically ill COVID-19 patients.

In addition to *CLEC12A*, we also identified the time-independent increased expression of *ACHE* in COVID-19 patients with a worse outcome. Like *CLEC12A*, *ACHE* affects the regulation of immune response intensity. The substrate of ACHE, acetylcholine (ACh) also acts as an anti-inflammatory molecule in addition to its function as a neurotransmitter. ACh can activate the cholinergic anti-inflammatory pathway (CAP) by binding to the α7 nicotinic acetylcholine receptor (α7 nAChR), which inhibits the TLR-activated NF-κB pathway and thus the expression of inflammatory cytokines such as IL-6, IL-8, and TNFα^31^. The observed increased expression of *ACHE* would lead to a decrease in ACh levels as it is metabolized, and thus ACHE might have a proinflammatory effect in COVID-19 patients leading to a worse outcome. Farsalinos et al*.* hypothesized that some clinical manifestations of COVID-19 (anosmia, cytokine storm, and thromboembolic complications) may also be associated with dysfunction of the nicotinic cholinergic system or CAP, but no clear evidence of a connection between COVID-19 severity and CAP has been described yet^32^. On the other hand, the increased *ACHE* expression in patients with a worse clinical outcome could be a manifestation of the observed increased differentiation of hematopoietic stem cells into erythrocytes since *ACHE* is primarily expressed in neuronal cells or erythrocytes. Nevertheless, the demonstrated potential of *ACHE* to predict the severity of COVID-19, especially in combination with *CLEC12A* holds great potential. The identified biomarkers *CLEC12A* and *ACHE* might both examine the ability of an individual to inhibit upstream activation of the NF-κB pathway, albeit by different mechanisms, and thus the ability to prevent an overwhelming immune response in COVID-19. It should be explicitly noted, that both patient groups received immunosuppressive drug treatment (e.g. dexamethasone) in a comparable measure. In principle, the increased expression of *ACHE* in COVID-19 non-survivors and the increased expression of *CLEC12A* in COVID-19 survivors could be due to either COVID-19 itself or might represent an unknown predisposing factor for the development of severe COVID-19. The significantly lower expression of *CLEC12A* and *ACHE* in healthy controls compared to the two COVID-19 patient groups suggests that the expression of these genes might be induced during severe COVID-19 and is probably not a predisposing factor. However, this finding still needs to be validated within larger groups of COVID-19 patients and healthy controls for a more robust evaluation.

Several publications characterize the blood transcriptome and host-response of COVID-19 patients, but their main focus is not the identification of biomarkers for risk stratification or outcome prediction^33–36^. So far, no study on blood transcriptome biomarkers for outcome prediction of COVID-19 has been published, but an interesting prospective validation study has been announced^37^. The presented results in this study might improve the ability to determine which COVID-19 patients are at high risk of death in the ICU based on intracellular transcription levels of two proteins. The ability of *ACHE* and *CLEC12A* to discriminate between a favorable and unfavorable outcome for COVID-19 patients showed better performance than that of existing biomarkers, like CRP and PCT independent of the sampling time, and better than the SOFA score at day 0. Comparison of our blood transcriptome biomarkers with other recently reported novel biomarkers for outcome prediction in COVID-19 patients using nasal swabs or ELISA assays will provide considerable value for patient stratification^38,39^. The main limitation of the presented study are the small sample sizes. To overcome some of the resulting drawbacks, the comparably small sample size of the RT-qPCR verification experiments should be increased to evaluate more accurately the classification performance of *CLEC12A* and *ACHE* biomarkers. In addition, the clinical utility also needs to be evaluated to determine whether early identification of COVID-19 patients in the ICU who are at high risk of mortality can improve outcomes. Given the recent development and approval of drugs such as Paxlovid or Molnupiravir that reduce mortality in COVID-19 patients when administered early, this issue is of great interest.

## Methods

### Study design

This study included COVID-19 patients of the first, second, and beginning third wave of the pandemic in Germany, who were treated on the ICU of University Hospital Essen between April 2020 and August 2021^40^. In addition, healthy individuals were included as controls. Study protocols were approved by the ethics committee of the medical faculty of the University Duisburg-Essen (17-7824-BO and 20-9216-BO). Patients and healthy controls were enrolled after detailed information and written informed consent. All experiments were performed in accordance with the approved protocols. Participating patients were assessed by the WHO Ordinal Scale of Clinical Improvement (score 0–10) and were grouped into two groups according to their disease severity and clinical outcome (worst rating during hospitalization): survivors (WHO 5–6) and non-survivors (WHO 10)^3^. All patients received a guideline conform COVID-19 therapy according to the valid AWMF guideline or the COVRIIN recommendations^41–43^. In total, 12 surviving patients with a WHO score of 5 or 6 (belonging to the moderate or severe disease group) and 12 non-surviving patients with a WHO score of 10 were matched according to age, gender, BMI, and SOFA-score at day 0 (Supplementary Table S1). For all 24 patients, blood samples were collected at admission to the ICU (day 0) and seven days after admission (day 7), resulting in a total sample size of n = 48 (day 0: n = 24 (survivor n = 12, non-survivor n = 12), day 7: n = 24 (survivor n = 12, non-survivor n = 12)). The same sample sizes apply to the clinical standard of care markers analyzed, namely CRP, SOFA, and PCT. Following identification of differentially transcribed genes with prognostic potential by RNA-Seq, a reverse transcription quantitative PCR (RT-qPCR) approach was established for clinical validation (Supplementary Figure S1). Additionally, RT-qPCR assays were performed with blood samples from independent healthy individuals (n = 13).

### RNA extraction

Blood of all patients was collected in PAXgene Blood RNA tubes (BD, Heidelberg, Germany), incubated at room temperature for 2 h to achieve complete lysis of blood cells, and frozen at − 80 °C until further processing. Before nucleic acid isolation, tubes were thawed at room temperature for 2 h. Nucleic acid isolation was performed using the QIAcube (Qiagen, Hilden, Germany) and the PAXgene blood miRNA kit according to the manufacturer’s protocol to extract gene-encoding mRNAs. On QIAcube, part A of the standard PAXgene blood miRNA protocol was used. Nucleic acids were eluted in 2 × 40 µL Buffer BR5. The quantity and quality of the isolated RNA was determined with a Qubit Fluorometer 3.0 (Life Technologies, California, USA) and a Fragment Analyzer (Agilent, Santa Clara, California, USA), respectively.

### Reverse transcription quantitative PCR

500 ng of total RNA was converted into cDNA using QuantiNovaTM Reverse Transcription Kit (QIAGEN, Hilden, Germany) according to the manufacturer’s protocol. The utilized kit includes a genomic DNA elimination step. Reverse transcription quantitative PCR (RT-qPCR) was performed using the LightCycler®480 (Roche, Mannheim, Germany). A 20 μL reaction contained 10 µl 2 × QuantiTect SYBR Green PCR Master Mix (QIAGEN, Hilden, Germany), 3 μL ddH_2_O, 6 μL cDNA template, and 2.5 μM of each primer. The primer sequences are provided in Supplementary Table S2. The following thermal conditions for amplification were applied: 95 °C for 15 min, followed by 45 cycles at 94 °C for 15 s, 60 °C for 30 s and 72 °C for 30 s, and final 72 °C for 5 min. Melting curves were obtained by slow heating (0.5 °C/s) at temperatures in the range of 65 to 95 °C. The 2^-ΔΔCT^ method was performed for relative quantification analysis using the housekeeping genes *PEX16* and *FPGS*^44^. For *PEX16*, the PCR product was analyzed using Fragment Analyzer (Agilent, Santa Clara, California, USA) to ensure that only a single PCR product was obtained (Supplementary Figure S6). Samples were excluded from RT-qPCR if less than 400 ng of RNA remained after NGS. This resulted in a reduced sample size of n = 33 (survivors n = 18 (day 0 n = 8, day 7 n = 10), non-survivors n = 15 (day 0 n = 9, day 7 n = 6).

### Preparation of NGS libraries and sequencing

Library preparation and sequencing were performed using 200 ng RNA with the TruSeq RNA library prep kit v2 (Illumina, San Diego, CA, USA), using a Biomek FXP liquid handling robot (Beckman Coulter, Brea, CA, USA). Sequencing of the libraries was performed with NextSeq2000 (Illumina, San Diego, CA, USA), resulting in an average of 50 million 50 bp single-end reads per sample.

### Differential gene expression analysis

Raw sequencing reads were processed with BBTools (bbduk.sh) to remove sequencing artifacts and poor-quality reads^45^. Reads were mapped against the human reference genome assembly GRCh38.80 using NextGenMap (v. 0.5.5) with default settings^46^. Downstream quantification of genes in raw read counts as well as in TPM (= transcripts per million mapped reads according to Wagner et al.^47^*.*) was carried out exclusively with uniquely mapped reads using Gencode annotation v22 (Ensembl release 80) with the python script “rpkmforgenes.py” by Ramskold et al*.* (available at https://bit.ly3262/QgQ^48^ . Identification of DEGs was done with the R package DEBrowser (v. 1.2.0) using the implemented DESeq2 method with raw read counts and default settings^49,50^. Genes were considered as differentially expressed between two conditions (12 biological replicates per condition) with an adjusted *p-*value (FDR, false discovery rate) ≤ 0.01 and a log_2_ fold change ≤  − 1 or ≥ 1. Boxplots were created with the R package ggpubr (available at https://github.com/kassambara/ggpubr). Volcano plots for the differential expression analysis were generated with VolcaNoseR^51^. The dimensions of the identified DEGs were reduced by principal component analysis (PCA), and the first three principal components were visualized in R with pca3d (v. 0.10.2). In addition, the biological functions of the DEGs were analyzed using g:Profiler with default settings (over-representation analysis, ORA), and pathways with an adjusted *p-*value ≤ 0.05 were considered significant^52^.

### Biomarker classification performance

DEGs of the surviving and non-surviving comparisons at day 0 and day 7 were compared using DiVenn (v. 2.0.0) to identify time-independent DEGs^53^. The time-independent DEGs were evaluated and ranked based on their ability to classify patients into survivors or non-survivors regardless of the time point. For this purpose, the non-normalized TPM value of the respective gene was used as a predictor for ROC curve analysis in R with pROC (v. 1.17.0.1)^54^. For improved classification power, two biomarkers were combined in a binomial generalized linear model (GLM) as proposed by Mazzara et al*.*^18^. The GLM has the following regression Eq. (1):

1 $${\text{f}}\left( {\text{X}} \right){ = }\beta_{{0}} { + }\beta_{{1}} \cdot {\text{log}}\left( {{\text{X}}_{{1}} { + 1}} \right){ + }\beta_{{2}} \cdot {\text{log(X}}_{{2}} { + 1)}$$

where β are the coefficients determined by the model and X the independent variables, which are the biomarker readouts (e.g. 2^−ΔΔCT^ (gene)). To predict the outcome probability of a patient, the following sigmoid function (2) was used:

2 $${\text{p}}\left( {\text{X}} \right){ = 1}/{\text{(1 + e}}^{{{\text{ - f}}\left( {\text{X}} \right)}} {)}$$

where p(X) is the probability that a patient survives. Differences between AUROC curves were tested for statistical significance with the "roc.test" function from the R package pROC using the bootstrap method and 10.000 permutations, while the remaining parameters were set to default values (one-sided test, α = 0.05).

### Detection of infectious microorganisms in blood plasma of patients

Library preparation and sequencing were carried out as previously described from 1 ng cell-free deoxyribonucleic acid (cfDNA) using the NEXTFLEX Cell Free DNA-Seq Library Prep Kit 2.0 (Perkin-Elmer, Waltham, MA) with a Biomek FXP liquid handling robot (Beckman Coulter, Brea, CA)^16^. Sequencing of the libraries was performed on a NextSeq2000 (Illumina, San Diego, CA), resulting in 25–30 million 100 bp single-end reads, on average, per sample. Bioinformatic processing and sepsis indicating quantifier (SIQ) score calculation were carried out as described before^16^.

## Supplementary Information

Supplementary Information 1.

Supplementary Information 2.

## Data Availability

The utilized raw RNA-Seq data is deposited in the sequence read archive (SRA) of the national center for biotechnology information (NCBI) under the following accession number: PRJN815981. All data are fully available without restriction.
