# Supplementary material for: Identification of two early blood biomarkers ACHE and CLEC12A for improved risk stratification of critically ill COVID-19 patients
Source: Sci Rep. 2023 Mar 16;13:4388. doi: 10.1038/s41598-023-30158-1 (PMC10019437; doi:10.1038/s41598-023-30158-1)
Supplement: Supplementary file 1 — Supplementary Information 1. [file 41598_2023_30158_MOESM1_ESM.docx]

**Supplementary Materials**

Identification of two early blood biomarkers *ACHE* and *CLEC12A* for improved risk stratification of critically ill COVID-19 patients

Simone Kattner^1^*, Jan Müller^2,3,4^*, Karolina Glanz^2^, Mehdi Manoochehri^2^, Caroline Sylvester^2^, Yevhen Vainshtein^2^, Marc Moritz Berger^1^, Thorsten Brenner^1+^, Kai Sohn^2+^

1 Department of Anesthesiology and Intensive Care Medicine, University Hospital Essen, University Duisburg-Essen, Essen, Germany

2 Innovation Field In-vitro Diagnostics, Fraunhofer Institute for Interfacial Engineering and Biotechnology IGB, Stuttgart, Germany

3 Center for Integrative Bioinformatics Vienna (CIBIV), Max Perutz Labs, University of Vienna and Medical University of Vienna, Vienna BioCenter, Vienna, Austria

4 Vienna BioCenter PhD Program, Doctoral School of the University of Vienna and Medical University of Vienna, Vienna, Austria

*Equal contribution

^+^Shared senior authorship and corresponding authors: Kai Sohn (kai.sohn@igb.fraunhofer.de), Thorsten Brenner (Thorsten.Brenner@uk-essen.de)


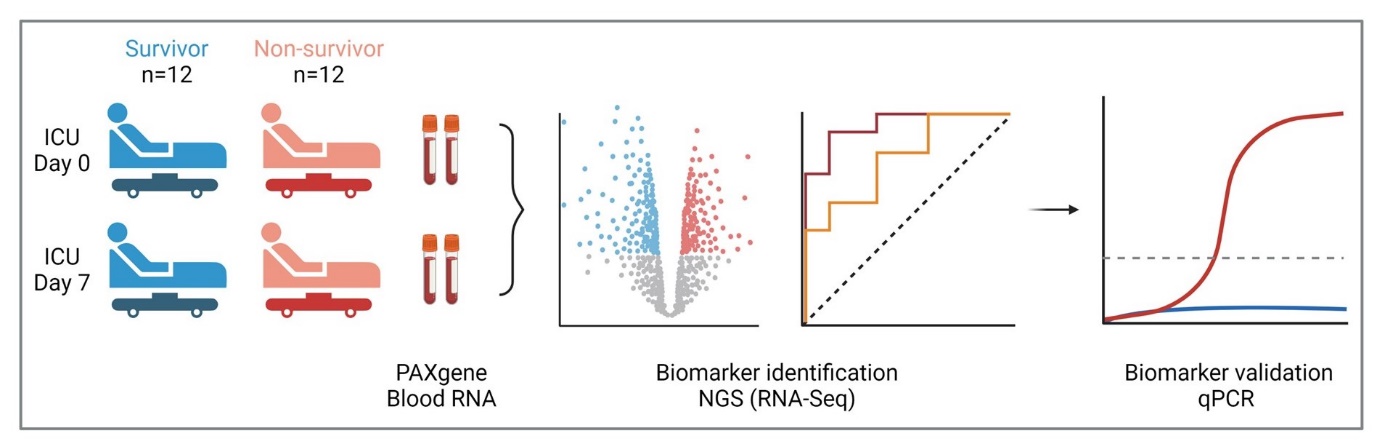


**Supplementary Figure S1 – Study design to identify biomarkers for survival prediction of COVID-19 patients at the ICU.** Twenty-four age-matched SARS-CoV-2-positive patients were enrolled in this study after admission to the ICU, of whom twelve survived and recovered. Blood samples were collected from each patient on day 0 (ICU admission) and after 7 days. 48 PAXgene blood samples were subsequently used for unbiased biomarker identification by RNA-seq. The identified putative biomarkers were further validated by RT-qPCR. Created with BioRender.com.


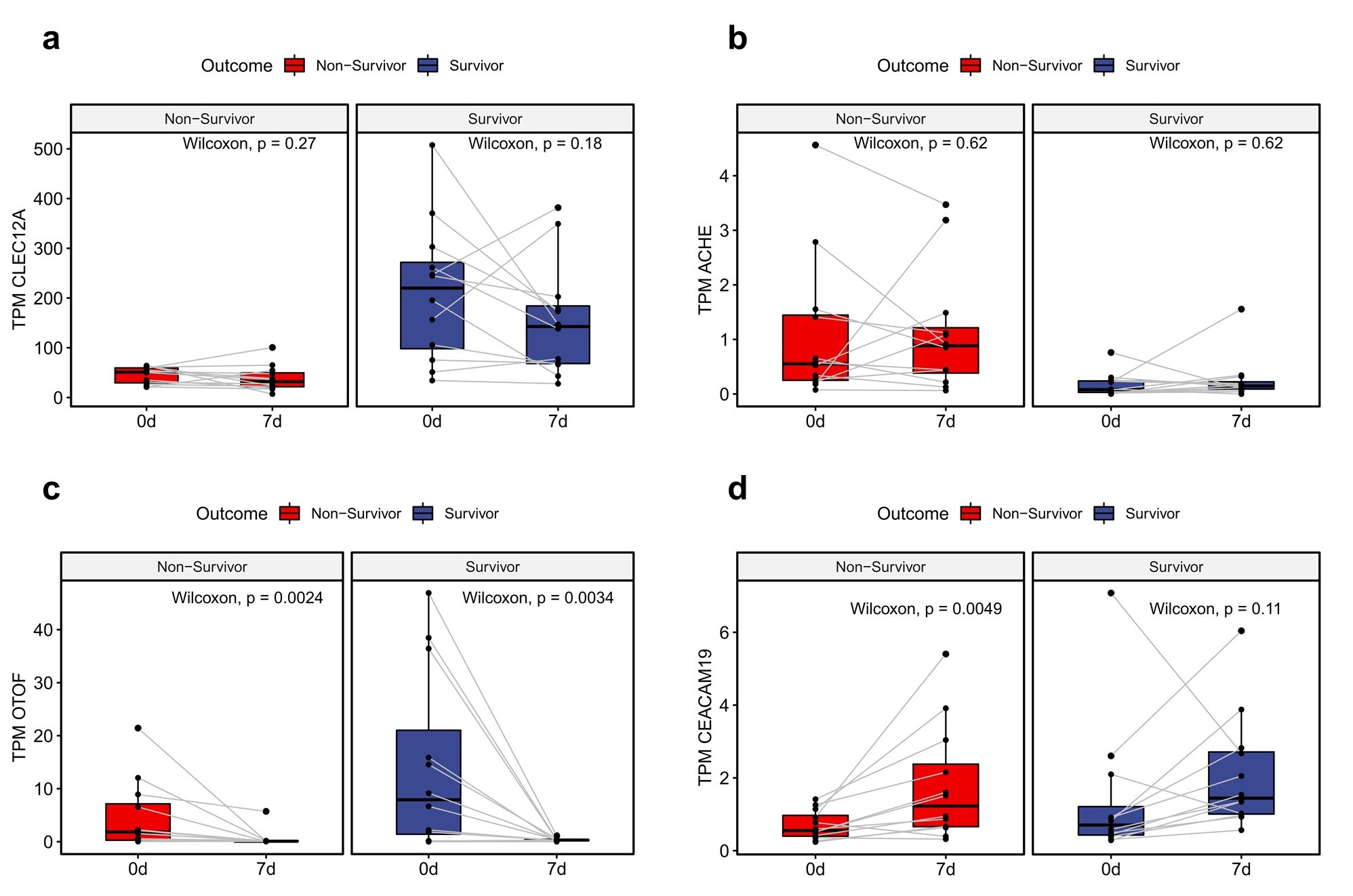


**Supplementary Figure S2 –** **Paired boxplots comparing expression levels of time independent genes, *CLEC12A* and *ACHE*, with time dependent genes, *OTOF* and *CEACAM19* for severe COVID-19 patients.** (a) Boxplots for *CLEC12A* expression levels in survivors or non-survivors. (b) Boxplots for *ACHE* expression levels in survivors or non‑survivors. (c) Boxplots for *OTOF* expression levels in survivors or non-survivors. (d) Boxplots for *CEACAM19* expression levels in survivors or non-survivors. Both sampling time points are shown separately. Differences between the means of the two outcome groups were assessed with a two-sided, paired Wilcoxon test separately for day 0 and day 7 with n=24 (survivor n=12, non-survivor n=12).


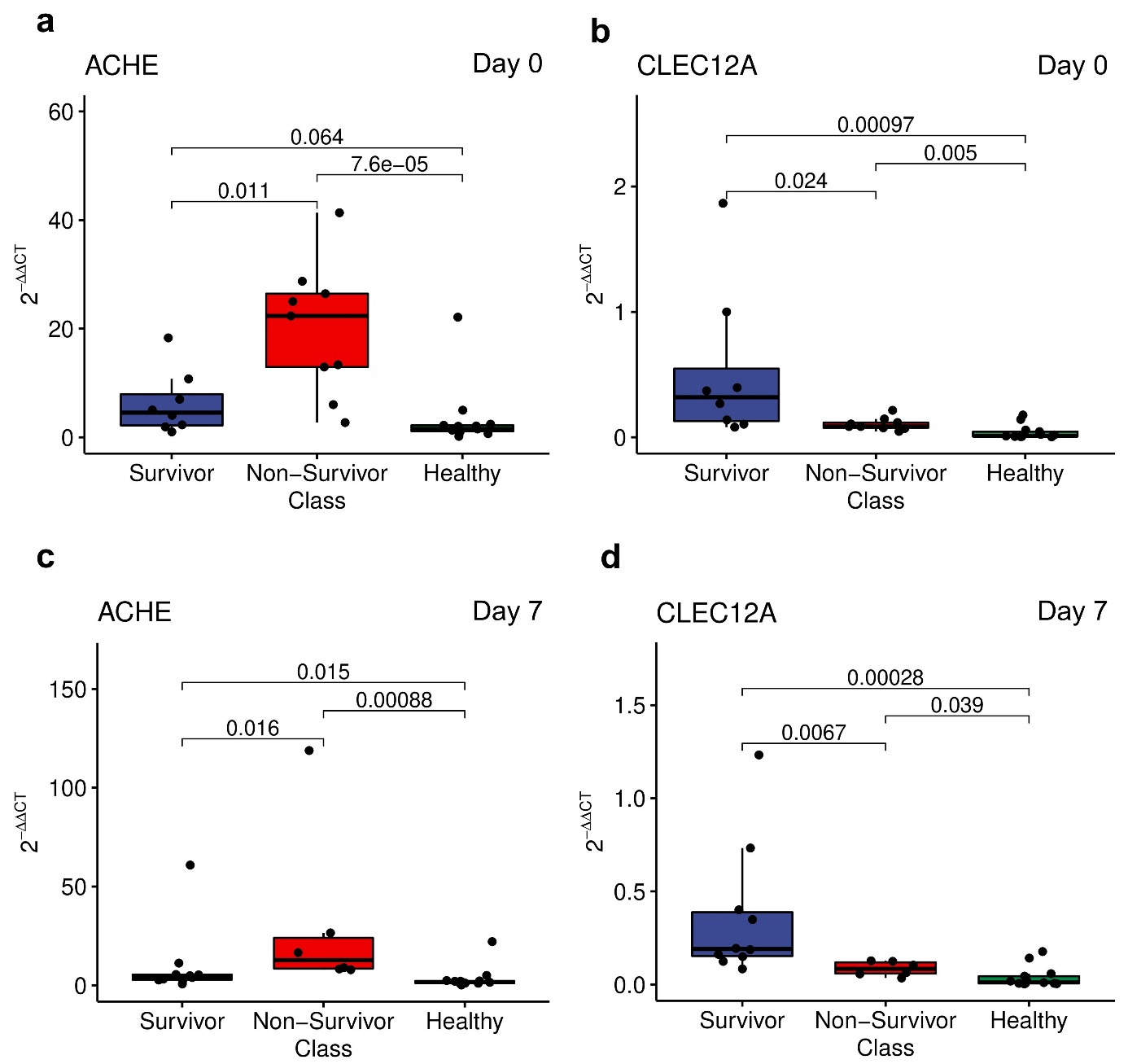


**Supplementary Figure S3 – Boxplots comparing expression levels of *CLEC12A* and *ACHE* at day 0 and day 7 between COVID-19 survivors, non-survivors, and independent healthy individuals.** (a) Boxplots for *ACHE* expression levels at day 0 (survivors n=8, non-survivors n=9). (b) Boxplots for *CLEC12A* expression levels at day 0 (survivors n=8, non-survivors n=9). (c) Boxplots for *ACHE* expression levels at day 7 (survivors n=10, non-survivors n=6). (d) Boxplots for *CLEC12A* expression levels at day 7 (survivors n=10, non-survivors n=6). Differences between means of two groups were determined separately using a two-tailed Wilcoxon test, and the corresponding p-value is given for each comparison. The same group of 13 independent healthy individuals was used for all comparisons in (a) - (d) and annotated sampling times do not apply to this group. Additionally, unequal variance t-tests were performed (COVID-19 non-survivors vs. COVID-19 survivors: p_ACHE,day0_=0.007, p_ACHE,day7_=0.005, p_CLEC12A,day0_=0.017, p_CLEC12A,day7_=0.001; healthy controls vs. COVID-19 survivors: p_ACHE,day0_=0.061, p_ACHE,day7_=0.012, p_CLEC12A,day0_=4.8⋅10^-5^, p_CLEC12A,day7_=7.6⋅10^-6^; healthy controls vs. COVID-19 non-survivors: p_ACHE,day0_=9.5⋅10^-6^, p_ACHE,day7_=4.8⋅10^-5^, p_CLEC12A,day0_=9.3⋅10^-4^, p_CLEC12A,day7_=0.007).


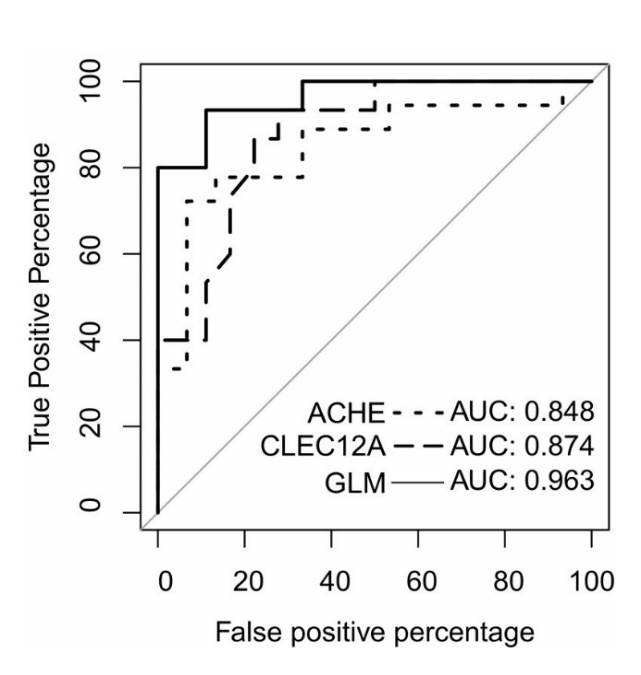


**Supplementary Figure S4 – Time independent patient classification with RT-qPCR data for *CLEC12A* and *ACHE*.** AUROC curve analysis for classification of COVID-19 patients into survivors n=18 (day 0 n=8, day 7 n=10) and non-survivors n=15 (day 0 n=9, day 7 n=6) for both time points combined. Individual biomarkers (*ACHE* and *CLEC12A*) and their combination in a generalized linear model (GLM) are shown.


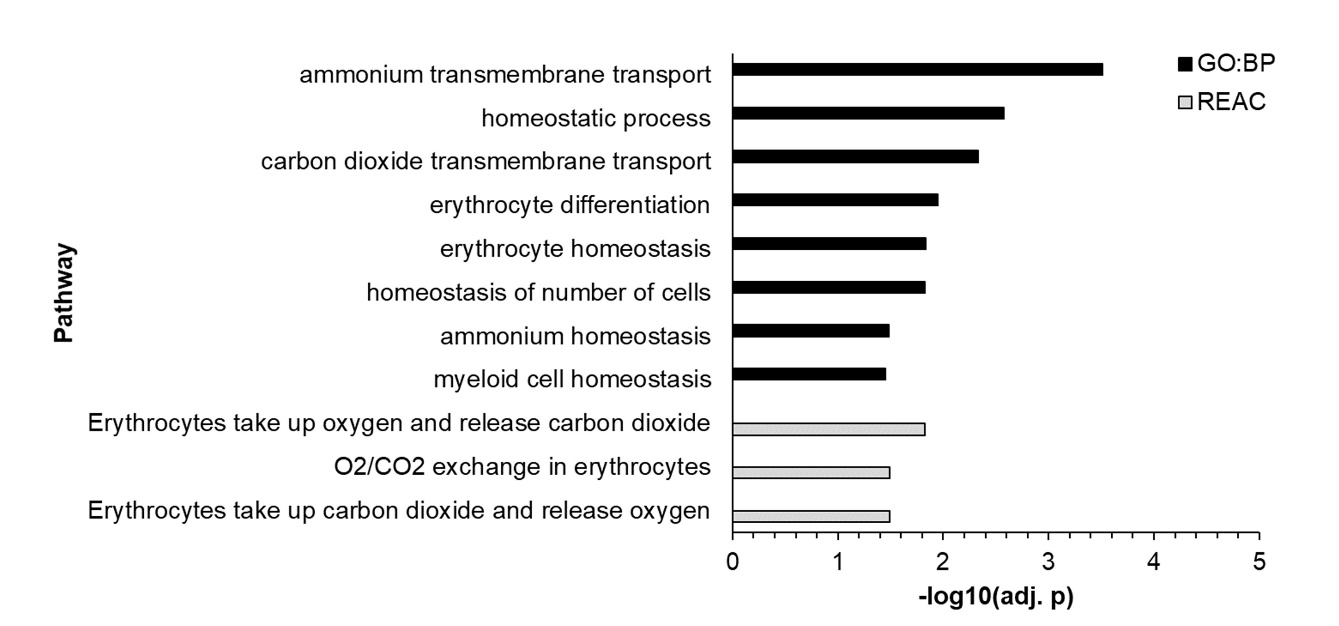


**Supplementary Figure S5 – Pathway enrichment analysis for 24 differentially expressed genes identified at both day 0 and day 7 between COVID-19 survivors and non-survivors.** GeneOntology (Biological Processes), KEGG, Reactome, and Wikipathways were used in this over-representation pathway analysis for the gene set. Only pathways with an adjusted p‑value ≤ 0.05 for enrichment are shown.

**Table S1 – Patient metadata of the study cohort at day 0.** Two-sided Wilcoxon test was used to test for the significant difference of means.

|  | **Non-survivors**  **(n=12)** | **Survivors**  **(n=12)** | **Patients combined**  **(n=24)** | **p-value (Wilcoxon comparison of means)** |
| --- | --- | --- | --- | --- |
| Age (mean±SD) [years] | 64.7±15.1 | 65.1±14.5 | 64.9±14.5 | 0.86 |
| BMI (mean±SD) | 27.8±5.9 | 26.6±6.9 | 27.3±6.2 | 0.53 |
| SOFA (mean±SD) | 4.9±2.3 | 3.2±1.9 | 4.0±2.3 | 0.10 |
| Gender ratio [male/female] | 10/2 | 9/3 | 19/5 | - |
| Immunosuppressive drug treatment [yes/no] | 11/1 | 10/2 | 21/3 | - |

**Table S2 – Details of the primers used in the RT-qPCR assay.** Primers for RT-qPCR analysis of the expression levels of *ACHE*, *CLEC12A,* and the reference genes *PEX16* and *FPGS* are given.

| Primer | Target | Amplicon size (nt) | Sequence (5'-3') | Strand | Binding location (hg19) |
| --- | --- | --- | --- | --- | --- |
| ACHE_fw | *ACHE* | 76 | GGGTGGTAGACGCTACAACC | + | chr7:100893891-100893912 |
| ACHE_rev |  |  | GTGCCCTCAAAACCTGGGTAT | - | chr7:100893947-100893967 |
| CLEC12A_fw | *CLEC12A* | 92 | TAGCCACCAAATTATGTCGTGAG | + | chr12:9979482-9979505 |
| CLEC12A_rev |  |  | GCTGTCCTTATGCCAAATCCATC | - | chr12:9980608-9980631 |
| FPGS_fw | *FPGS* | 115 | CCGAGGTTCGAGTCTTGCTC | + | chr9:127810035-127810055 |
| FPGS_rev |  |  | TCTGTCAGGTTAGGGCAGAAG | - | chr9:127810967-127810988 |
| PEX16_fw | *PEX16* | 82 | GTGCGGGGCTTCAGTTACC | + | chr11:45917708-45917727 |
| PEX16_rev |  |  | GGTTAGAGGCAGAGTACACCA | - | spanning exon junction of exon 2 and 3 |


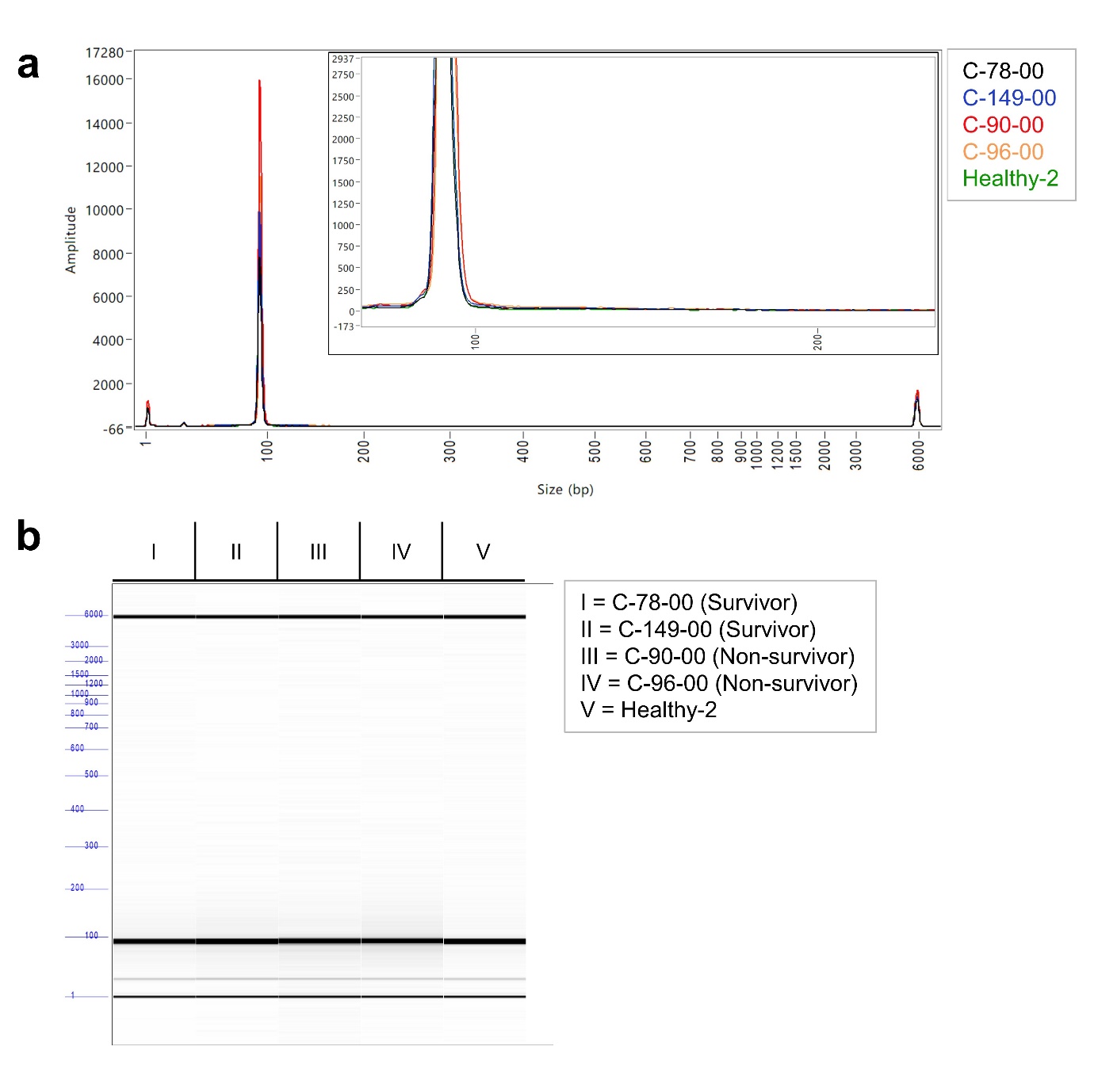


**Supplementary Figure S6 – Analysis of PCR products obtained with the PEX16 primer pair.** (a) Electropherogram showing signal amplitude (relative fluorescence) versus fragment size (retention time). Insert with lower signal amplitude for the size range 50-250 bp. (b) Gel-like visualization of the data shown in (a). Five samples from all three patient groups presented in the manuscript (COVID-19: survivors (n=2), non-survivors (n=2), and healthy (n=1)) were analyzed. Sample identifiers are provided in legends.

**Table S3 – ROC curve analysis results for the identified 24 genes that are differentially expressed on day 0 and day 7.** The area under the ROC (AUROC) curve was calculated with data from both time points, day 0 and day 7.

| ENSEMBL gene ID | Gene symbol | Direction  (High in) | AUROC | Lower Limit  95% CI | Upper Limit  95% CI |
| --- | --- | --- | --- | --- | --- |
| ENSG00000172322 | *CLEC12A* | Survivors | 0.908 | 0.822 | 0.994 |
| ENSG00000188672 | *RHCE* | Non-survivors | 0.875 | 0.774 | 0.976 |
| ENSG00000256660 | *CLEC12B* | Survivors | 0.863 | 0.754 | 0.972 |
| ENSG00000087085 | *ACHE* | Non-survivors | 0.837 | 0.722 | 0.951 |
| ENSG00000182795 | *C1orf116* | Non-survivors | 0.833 | 0.714 | 0.953 |
| ENSG00000170180 | *GYPA* | Non-survivors | 0.816 | 0.693 | 0.939 |
| ENSG00000178297 | *TMPRSS9* | Non-survivors | 0.816 | 0.695 | 0.937 |
| ENSG00000112077 | *RHAG* | Non-survivors | 0.806 | 0.677 | 0.935 |
| ENSG00000163554 | *SPTA1* | Non-survivors | 0.792 | 0.651 | 0.932 |
| ENSG00000112212 | *TSPO2* | Non-survivors | 0.788 | 0.656 | 0.920 |
| ENSG00000108309 | *RUNDC3A* | Non-survivors | 0.786 | 0.655 | 0.918 |
| ENSG00000076864 | *RAP1GAP* | Non-survivors | 0.776 | 0.636 | 0.916 |
| ENSG00000008441 | *NFIX* | Non-survivors | 0.773 | 0.642 | 0.904 |
| ENSG00000133069 | *TMCC2* | Non-survivors | 0.762 | 0.624 | 0.900 |
| ENSG00000187997 | *C17orf99* | Non-survivors | 0.743 | 0.602 | 0.885 |
| ENSG00000240583 | *AQP1* | Non-survivors | 0.740 | 0.593 | 0.886 |
| ENSG00000105610 | *KLF1* | Non-survivors | 0.736 | 0.592 | 0.880 |
| ENSG00000166793 | *YPEL4* | Non-survivors | 0.691 | 0.538 | 0.844 |
| ENSG00000197465 | *GYPE* | Non-survivors | 0.682 | 0.529 | 0.836 |
| ENSG00000117394 | *SLC2A1* | Non-survivors | 0.677 | 0.515 | 0.840 |
| ENSG00000183111 | *ARHGEF37* | Non-survivors | 0.665 | 0.506 | 0.824 |
| ENSG00000143479 | *DYRK3* | Non-survivors | 0.663 | 0.496 | 0.831 |
| ENSG00000130202 | *NECTIN2* | Non-survivors | 0.656 | 0.493 | 0.820 |
| ENSG00000106327 | *TFR2* | Non-survivors | 0.630 | 0.469 | 0.792 |

**Table S4 – Full list of all differentially expressed genes on day 0 and day 7.** Genes were considered as differentially expressed with a false discovery rate (FDR) ≤ 0.01 and a fold change ≤ −2 or ≥ 2.

| ENSEMBL gene ID | Gene symbol | Time | Direction  (High in) | log_2_(FC) | FDR |
| --- | --- | --- | --- | --- | --- |
| ENSG00000129673 | *AANAT* | Day 7 | Non-Survivor | 2.02 | 2.37E-03 |
| ENSG00000115657 | *ABCB6* | Day 7 | Non-Survivor | 2.22 | 1.93E-08 |
| ENSG00000166016 | *ABTB2* | Day 0 | Non-Survivor | 1.38 | 6.51E-03 |
| ENSG00000176244 | *ACBD7* | Day 7 | Non-Survivor | 2.32 | 7.11E-03 |
| ENSG00000087085 | *ACHE* | Day 0 | Non-Survivor | 3.51 | 1.30E-03 |
| ENSG00000087085 | *ACHE* | Day 7 | Non-Survivor | 2.88 | 9.53E-03 |
| ENSG00000147174 | *ACRC* | Day 7 | Non-Survivor | 1.46 | 5.92E-04 |
| ENSG00000123612 | *ACVR1C* | Day 7 | Survivor | -1.59 | 1.19E-03 |
| ENSG00000075340 | *ADD2* | Day 7 | Non-Survivor | 1.99 | 6.94E-03 |
| ENSG00000121753 | *ADGRB2* | Day 7 | Survivor | -1.59 | 3.56E-03 |
| ENSG00000159346 | *ADIPOR1* | Day 0 | Non-Survivor | 1.39 | 3.08E-03 |
| ENSG00000204305 | *AGER* | Day 7 | Non-Survivor | 1.30 | 4.21E-03 |
| ENSG00000169877 | *AHSP* | Day 0 | Non-Survivor | 3.05 | 5.79E-04 |
| ENSG00000140057 | *AK7* | Day 7 | Non-Survivor | 2.65 | 9.65E-03 |
| ENSG00000204673 | *AKT1S1* | Day 7 | Non-Survivor | 1.13 | 2.37E-03 |
| ENSG00000137760 | *ALKBH8* | Day 7 | Survivor | -1.09 | 1.99E-03 |
| ENSG00000136383 | *ALPK3* | Day 7 | Non-Survivor | 1.97 | 2.65E-04 |
| ENSG00000029534 | *ANK1* | Day 0 | Non-Survivor | 2.21 | 4.04E-04 |
| ENSG00000160117 | *ANKLE1* | Day 7 | Non-Survivor | 1.23 | 1.92E-03 |
| ENSG00000165887 | *ANKRD2* | Day 7 | Non-Survivor | 2.67 | 5.06E-03 |
| ENSG00000164236 | *ANKRD33B* | Day 0 | Non-Survivor | 1.03 | 9.21E-03 |
| ENSG00000164512 | *ANKRD55* | Day 7 | Non-Survivor | 1.89 | 3.56E-03 |
| ENSG00000105290 | *APLP1* | Day 7 | Non-Survivor | 2.59 | 7.15E-04 |
| ENSG00000240583 | *AQP1* | Day 0 | Non-Survivor | 2.46 | 1.30E-03 |
| ENSG00000240583 | *AQP1* | Day 7 | Non-Survivor | 2.37 | 2.53E-03 |
| ENSG00000081181 | *ARG2* | Day 7 | Non-Survivor | 1.41 | 1.14E-03 |
| ENSG00000074964 | *ARHGEF10L* | Day 7 | Survivor | -1.43 | 4.03E-03 |
| ENSG00000196914 | *ARHGEF12* | Day 0 | Non-Survivor | 1.16 | 4.52E-03 |
| ENSG00000183111 | *ARHGEF37* | Day 0 | Non-Survivor | 2.01 | 6.46E-03 |
| ENSG00000183111 | *ARHGEF37* | Day 7 | Non-Survivor | 2.35 | 4.69E-03 |
| ENSG00000111339 | *ART4* | Day 7 | Non-Survivor | 3.93 | 6.03E-04 |
| ENSG00000100325 | *ASCC2* | Day 0 | Non-Survivor | 1.23 | 3.01E-03 |
| ENSG00000198925 | *ATG9A* | Day 7 | Non-Survivor | 1.13 | 2.47E-03 |
| ENSG00000129244 | *ATP1B2* | Day 7 | Non-Survivor | 3.12 | 1.63E-03 |
| ENSG00000124172 | *ATP5E* | Day 7 | Non-Survivor | 1.10 | 2.83E-03 |
| ENSG00000180389 | *ATP5EP2* | Day 7 | Non-Survivor | 1.10 | 4.79E-03 |
| ENSG00000107262 | *BAG1* | Day 0 | Non-Survivor | 1.29 | 2.67E-03 |
| ENSG00000187244 | *BCAM* | Day 0 | Non-Survivor | 3.21 | 4.27E-03 |
| ENSG00000171552 | *BCL2L1* | Day 0 | Non-Survivor | 1.81 | 1.45E-03 |
| ENSG00000242252 | *BGLAP* | Day 7 | Non-Survivor | 1.40 | 2.76E-03 |
| ENSG00000152785 | *BMP3* | Day 7 | Survivor | -3.13 | 7.18E-03 |
| ENSG00000104765 | *BNIP3L* | Day 0 | Non-Survivor | 1.10 | 2.51E-03 |
| ENSG00000163141 | *BNIPL* | Day 7 | Non-Survivor | 1.90 | 2.60E-05 |
| ENSG00000172331 | *BPGM* | Day 0 | Non-Survivor | 2.28 | 3.23E-04 |
| ENSG00000151136 | *BTBD11* | Day 7 | Survivor | -1.24 | 1.84E-03 |
| ENSG00000166780 | *C16orf45* | Day 7 | Non-Survivor | 1.12 | 4.57E-03 |
| ENSG00000187997 | *C17orf99* | Day 0 | Non-Survivor | 2.18 | 8.48E-03 |
| ENSG00000187997 | *C17orf99* | Day 7 | Non-Survivor | 2.46 | 6.74E-03 |
| ENSG00000182795 | *C1orf116* | Day 0 | Non-Survivor | 2.21 | 5.01E-03 |
| ENSG00000182795 | *C1orf116* | Day 7 | Non-Survivor | 2.93 | 2.60E-05 |
| ENSG00000143443 | *C1orf56* | Day 7 | Non-Survivor | 1.15 | 1.89E-04 |
| ENSG00000221843 | *C2orf16* | Day 7 | Survivor | -1.24 | 5.50E-03 |
| ENSG00000182600 | *C2orf82* | Day 7 | Non-Survivor | 1.82 | 2.40E-03 |
| ENSG00000181577 | *C6orf223* | Day 7 | Non-Survivor | 3.89 | 1.47E-03 |
| ENSG00000185955 | *C7orf61* | Day 7 | Non-Survivor | 2.44 | 4.70E-04 |
| ENSG00000253250 | *C8orf88* | Day 7 | Non-Survivor | 2.39 | 1.70E-06 |
| ENSG00000188959 | *C9orf152* | Day 7 | Non-Survivor | 3.02 | 7.15E-04 |
| ENSG00000135045 | *C9orf40* | Day 7 | Non-Survivor | 1.19 | 9.69E-04 |
| ENSG00000136819 | *C9orf78* | Day 0 | Non-Survivor | 1.48 | 8.94E-03 |
| ENSG00000133742 | *CA1* | Day 0 | Non-Survivor | 4.31 | 2.09E-04 |
| ENSG00000104267 | *CA2* | Day 7 | Non-Survivor | 1.95 | 3.31E-03 |
| ENSG00000164879 | *CA3* | Day 7 | Non-Survivor | 2.26 | 9.17E-03 |
| ENSG00000100314 | *CABP7* | Day 7 | Non-Survivor | 1.42 | 4.38E-03 |
| ENSG00000141837 | *CACNA1A* | Day 7 | Non-Survivor | 1.53 | 4.70E-03 |
| ENSG00000006116 | *CACNG3* | Day 7 | Non-Survivor | 4.66 | 9.68E-04 |
| ENSG00000255221 | *CARD17* | Day 7 | Non-Survivor | 1.50 | 7.25E-03 |
| ENSG00000118307 | *CASC1* | Day 7 | Non-Survivor | 1.36 | 6.12E-03 |
| ENSG00000159588 | *CCDC17* | Day 7 | Non-Survivor | 1.83 | 2.27E-03 |
| ENSG00000119636 | *CCDC176* | Day 0 | Non-Survivor | 1.87 | 6.02E-04 |
| ENSG00000130783 | *CCDC62* | Day 7 | Non-Survivor | 1.18 | 8.68E-03 |
| ENSG00000101331 | *CCM2L* | Day 7 | Non-Survivor | 2.58 | 9.17E-03 |
| ENSG00000272398 | *CD24* | Day 0 | Non-Survivor | 1.43 | 7.07E-03 |
| ENSG00000120217 | *CD274* | Day 7 | Non-Survivor | 2.56 | 5.92E-04 |
| ENSG00000039068 | *CDH1* | Day 7 | Non-Survivor | 2.80 | 1.99E-03 |
| ENSG00000178404 | *CEP295NL* | Day 7 | Non-Survivor | 1.46 | 5.40E-03 |
| ENSG00000013297 | *CLDN11* | Day 7 | Non-Survivor | 1.68 | 6.72E-03 |
| ENSG00000213937 | *CLDN9* | Day 7 | Non-Survivor | 1.24 | 9.19E-03 |
| ENSG00000172322 | *CLEC12A* | Day 0 | Survivor | -1.47 | 5.34E-04 |
| ENSG00000172322 | *CLEC12A* | Day 7 | Survivor | -1.49 | 1.70E-03 |
| ENSG00000256660 | *CLEC12B* | Day 0 | Survivor | -1.74 | 2.45E-03 |
| ENSG00000256660 | *CLEC12B* | Day 7 | Survivor | -1.51 | 8.71E-03 |
| ENSG00000157322 | *CLEC18A* | Day 7 | Non-Survivor | 2.13 | 3.51E-03 |
| ENSG00000150048 | *CLEC1A* | Day 7 | Non-Survivor | 1.73 | 5.91E-03 |
| ENSG00000120885 | *CLU* | Day 7 | Non-Survivor | 1.88 | 1.92E-03 |
| ENSG00000166091 | *CMTM5* | Day 7 | Non-Survivor | 1.72 | 3.48E-03 |
| ENSG00000115649 | *CNPPD1* | Day 7 | Non-Survivor | 1.01 | 9.97E-03 |
| ENSG00000134871 | *COL4A2* | Day 7 | Non-Survivor | 3.05 | 2.92E-03 |
| ENSG00000114270 | *COL7A1* | Day 7 | Non-Survivor | 1.60 | 1.99E-03 |
| ENSG00000145244 | *CORIN* | Day 7 | Non-Survivor | 1.74 | 9.65E-03 |
| ENSG00000106034 | *CPED1* | Day 7 | Survivor | -1.39 | 3.17E-03 |
| ENSG00000157613 | *CREB3L1* | Day 7 | Non-Survivor | 4.33 | 4.66E-03 |
| ENSG00000103316 | *CRYM* | Day 7 | Non-Survivor | 2.84 | 3.69E-03 |
| ENSG00000162438 | *CTRC* | Day 7 | Non-Survivor | 1.54 | 2.83E-03 |
| ENSG00000196188 | *CTSE* | Day 0 | Non-Survivor | 2.82 | 5.79E-04 |
| ENSG00000142544 | *CTU1* | Day 7 | Non-Survivor | 1.37 | 2.48E-03 |
| ENSG00000140465 | *CYP1A1* | Day 7 | Non-Survivor | 2.10 | 2.75E-03 |
| ENSG00000155016 | *CYP2U1* | Day 7 | Survivor | -1.12 | 4.79E-03 |
| ENSG00000113758 | *DBN1* | Day 7 | Non-Survivor | 1.46 | 2.03E-03 |
| ENSG00000198876 | *DCAF12* | Day 0 | Non-Survivor | 1.42 | 2.55E-03 |
| ENSG00000213722 | *DDAH2* | Day 7 | Non-Survivor | 1.56 | 4.70E-03 |
| ENSG00000158856 | *DMTN* | Day 0 | Non-Survivor | 1.76 | 8.38E-03 |
| ENSG00000116675 | *DNAJC6* | Day 0 | Non-Survivor | 2.09 | 7.02E-03 |
| ENSG00000130158 | *DOCK6* | Day 7 | Non-Survivor | 1.47 | 7.25E-03 |
| ENSG00000159625 | *DRC7* | Day 7 | Non-Survivor | 2.49 | 4.54E-03 |
| ENSG00000143479 | *DYRK3* | Day 0 | Non-Survivor | 2.21 | 5.03E-03 |
| ENSG00000143479 | *DYRK3* | Day 7 | Non-Survivor | 2.06 | 8.40E-03 |
| ENSG00000143369 | *ECM1* | Day 7 | Non-Survivor | 1.98 | 9.84E-05 |
| ENSG00000172638 | *EFEMP2* | Day 7 | Non-Survivor | 1.32 | 2.14E-03 |
| ENSG00000172889 | *EGFL7* | Day 7 | Non-Survivor | 1.68 | 2.83E-03 |
| ENSG00000120738 | *EGR1* | Day 7 | Non-Survivor | 1.18 | 9.95E-03 |
| ENSG00000024422 | *EHD2* | Day 7 | Non-Survivor | 1.77 | 3.69E-03 |
| ENSG00000125037 | *EMC3* | Day 7 | Non-Survivor | 1.25 | 5.99E-03 |
| ENSG00000166947 | *EPB42* | Day 0 | Non-Survivor | 2.87 | 5.79E-04 |
| ENSG00000151491 | *EPS8* | Day 7 | Survivor | -1.72 | 8.24E-03 |
| ENSG00000149564 | *ESAM* | Day 7 | Non-Survivor | 1.43 | 5.25E-03 |
| ENSG00000130201 | *EXOC3L2* | Day 7 | Non-Survivor | 2.20 | 8.86E-05 |
| ENSG00000178896 | *EXOSC4* | Day 7 | Non-Survivor | 1.88 | 3.83E-04 |
| ENSG00000178752 | *FAM132B* | Day 7 | Non-Survivor | 3.95 | 2.65E-04 |
| ENSG00000124098 | *FAM210B* | Day 0 | Non-Survivor | 2.12 | 5.79E-04 |
| ENSG00000042062 | *FAM65C* | Day 7 | Non-Survivor | 2.12 | 2.70E-03 |
| ENSG00000147689 | *FAM83A* | Day 0 | Non-Survivor | 4.65 | 1.30E-03 |
| ENSG00000182118 | *FAM89A* | Day 7 | Non-Survivor | 1.13 | 5.45E-04 |
| ENSG00000116661 | *FBXO2* | Day 7 | Non-Survivor | 2.51 | 2.52E-03 |
| ENSG00000100225 | *FBXO7* | Day 0 | Non-Survivor | 1.40 | 7.02E-03 |
| ENSG00000066926 | *FECH* | Day 0 | Non-Survivor | 2.12 | 6.93E-04 |
| ENSG00000126262 | *FFAR2* | Day 7 | Non-Survivor | 1.61 | 8.52E-03 |
| ENSG00000185897 | *FFAR3* | Day 7 | Non-Survivor | 2.40 | 4.57E-03 |
| ENSG00000180263 | *FGD6* | Day 7 | Survivor | -1.30 | 4.70E-04 |
| ENSG00000107831 | *FGF8* | Day 7 | Non-Survivor | 2.96 | 8.29E-03 |
| ENSG00000115641 | *FHL2* | Day 7 | Non-Survivor | 1.91 | 2.37E-03 |
| ENSG00000139914 | *FITM1* | Day 7 | Non-Survivor | 1.55 | 2.27E-03 |
| ENSG00000119782 | *FKBP1B* | Day 7 | Non-Survivor | 2.10 | 2.76E-03 |
| ENSG00000004478 | *FKBP4* | Day 7 | Non-Survivor | 1.69 | 1.92E-03 |
| ENSG00000154803 | *FLCN* | Day 7 | Non-Survivor | 1.02 | 1.72E-03 |
| ENSG00000115226 | *FNDC4* | Day 7 | Non-Survivor | 3.65 | 8.72E-03 |
| ENSG00000087086 | *FTL* | Day 7 | Non-Survivor | 1.31 | 7.58E-03 |
| ENSG00000102145 | *GATA1* | Day 7 | Non-Survivor | 2.11 | 4.95E-03 |
| ENSG00000162645 | *GBP2* | Day 7 | Non-Survivor | 1.48 | 4.70E-03 |
| ENSG00000084734 | *GCKR* | Day 7 | Non-Survivor | 3.24 | 1.89E-04 |
| ENSG00000176928 | *GCNT4* | Day 7 | Survivor | -1.49 | 3.86E-03 |
| ENSG00000130513 | *GDF15* | Day 7 | Non-Survivor | 2.55 | 1.67E-04 |
| ENSG00000099998 | *GGT5* | Day 7 | Non-Survivor | 2.45 | 2.81E-03 |
| ENSG00000134812 | *GIF* | Day 7 | Non-Survivor | 1.65 | 6.58E-03 |
| ENSG00000111087 | *GLI1* | Day 7 | Non-Survivor | 1.73 | 3.18E-06 |
| ENSG00000182512 | *GLRX5* | Day 0 | Non-Survivor | 2.15 | 3.23E-04 |
| ENSG00000088053 | *GP6* | Day 7 | Non-Survivor | 1.35 | 6.58E-03 |
| ENSG00000169704 | *GP9* | Day 7 | Non-Survivor | 1.43 | 6.64E-03 |
| ENSG00000173264 | *GPR137* | Day 7 | Non-Survivor | 1.09 | 3.19E-03 |
| ENSG00000170075 | *GPR37L1* | Day 7 | Non-Survivor | 1.52 | 3.69E-03 |
| ENSG00000139572 | *GPR84* | Day 7 | Non-Survivor | 2.40 | 3.17E-03 |
| ENSG00000170180 | *GYPA* | Day 0 | Non-Survivor | 4.25 | 2.95E-04 |
| ENSG00000170180 | *GYPA* | Day 7 | Non-Survivor | 3.35 | 4.03E-03 |
| ENSG00000250361 | *GYPB* | Day 0 | Non-Survivor | 3.15 | 5.79E-04 |
| ENSG00000197465 | *GYPE* | Day 0 | Non-Survivor | 2.38 | 2.15E-03 |
| ENSG00000197465 | *GYPE* | Day 7 | Non-Survivor | 2.27 | 5.99E-03 |
| ENSG00000063854 | *HAGH* | Day 0 | Non-Survivor | 1.34 | 4.63E-03 |
| ENSG00000206172 | *HBA1* | Day 0 | Non-Survivor | 2.37 | 1.61E-03 |
| ENSG00000244734 | *HBB* | Day 0 | Non-Survivor | 2.36 | 4.63E-03 |
| ENSG00000223609 | *HBD* | Day 0 | Non-Survivor | 2.76 | 9.12E-04 |
| ENSG00000206177 | *HBM* | Day 7 | Non-Survivor | 2.85 | 2.76E-03 |
| ENSG00000255398 | *HCAR3* | Day 7 | Non-Survivor | 1.46 | 5.91E-03 |
| ENSG00000048052 | *HDAC9* | Day 7 | Survivor | -1.84 | 3.87E-04 |
| ENSG00000136929 | *HEMGN* | Day 0 | Non-Survivor | 2.22 | 4.27E-03 |
| ENSG00000188175 | *HEPACAM2* | Day 0 | Non-Survivor | 2.79 | 1.35E-03 |
| ENSG00000256269 | *HMBS* | Day 7 | Non-Survivor | 2.14 | 1.96E-03 |
| ENSG00000051128 | *HOMER3* | Day 7 | Non-Survivor | 1.46 | 3.72E-03 |
| ENSG00000134489 | *HRH4* | Day 0 | Non-Survivor | 2.47 | 5.79E-03 |
| ENSG00000099377 | *HSD3B7* | Day 7 | Non-Survivor | 2.20 | 7.89E-04 |
| ENSG00000106211 | *HSPB1* | Day 7 | Non-Survivor | 1.88 | 3.61E-03 |
| ENSG00000260325 | *HSPB9* | Day 7 | Non-Survivor | 1.34 | 3.28E-03 |
| ENSG00000090339 | *ICAM1* | Day 7 | Non-Survivor | 1.40 | 3.44E-03 |
| ENSG00000204010 | *IFIT1B* | Day 0 | Non-Survivor | 3.51 | 5.79E-04 |
| ENSG00000073792 | *IGF2BP2* | Day 0 | Non-Survivor | 1.62 | 8.17E-03 |
| ENSG00000188263 | *IL17REL* | Day 7 | Non-Survivor | 2.60 | 1.99E-03 |
| ENSG00000197272 | *IL27* | Day 7 | Non-Survivor | 2.30 | 2.55E-04 |
| ENSG00000139269 | *INHBE* | Day 7 | Non-Survivor | 2.56 | 1.19E-03 |
| ENSG00000248099 | *INSL3* | Day 7 | Non-Survivor | 2.04 | 4.03E-03 |
| ENSG00000185507 | *IRF7* | Day 7 | Non-Survivor | 1.25 | 5.45E-04 |
| ENSG00000005961 | *ITGA2B* | Day 7 | Non-Survivor | 2.03 | 5.45E-04 |
| ENSG00000135424 | *ITGA7* | Day 7 | Non-Survivor | 1.83 | 2.37E-03 |
| ENSG00000115221 | *ITGB6* | Day 7 | Non-Survivor | 3.22 | 2.75E-03 |
| ENSG00000197256 | *KANK2* | Day 0 | Non-Survivor | 2.68 | 4.04E-04 |
| ENSG00000176076 | *KCNE5* | Day 7 | Non-Survivor | 2.23 | 2.34E-03 |
| ENSG00000197993 | *KEL* | Day 0 | Non-Survivor | 1.96 | 8.17E-03 |
| ENSG00000140859 | *KIFC3* | Day 7 | Non-Survivor | 1.21 | 7.19E-03 |
| ENSG00000105610 | *KLF1* | Day 0 | Non-Survivor | 2.77 | 8.72E-04 |
| ENSG00000105610 | *KLF1* | Day 7 | Non-Survivor | 2.66 | 2.53E-03 |
| ENSG00000172059 | *KLF11* | Day 7 | Survivor | -1.32 | 1.19E-03 |
| ENSG00000104341 | *LAPTM4B* | Day 7 | Non-Survivor | 1.32 | 3.19E-03 |
| ENSG00000115850 | *LCT* | Day 7 | Non-Survivor | 3.20 | 6.12E-03 |
| ENSG00000183722 | *LHFP* | Day 7 | Non-Survivor | 2.45 | 2.37E-03 |
| ENSG00000163898 | *LIPH* | Day 7 | Non-Survivor | 1.79 | 3.03E-03 |
| ENSG00000173239 | *LIPM* | Day 7 | Non-Survivor | 2.56 | 2.48E-03 |
| ENSG00000176809 | *LRRC37A3* | Day 7 | Non-Survivor | 1.14 | 5.40E-03 |
| ENSG00000204421 | *LY6G6C* | Day 7 | Non-Survivor | 2.32 | 5.30E-03 |
| ENSG00000104903 | *LYL1* | Day 7 | Non-Survivor | 1.60 | 2.74E-03 |
| ENSG00000172264 | *MACROD2* | Day 7 | Survivor | -2.80 | 3.43E-03 |
| ENSG00000166963 | *MAP1A* | Day 7 | Non-Survivor | 1.28 | 2.74E-03 |
| ENSG00000188130 | *MAPK12* | Day 7 | Non-Survivor | 1.26 | 4.95E-03 |
| ENSG00000008735 | *MAPK8IP2* | Day 7 | Non-Survivor | 1.71 | 9.65E-03 |
| ENSG00000173926 | *MARCH3* | Day 7 | Non-Survivor | 1.60 | 7.15E-04 |
| ENSG00000165406 | *MARCH8* | Day 0 | Non-Survivor | 1.18 | 2.51E-03 |
| ENSG00000090674 | *MCOLN1* | Day 7 | Non-Survivor | 1.30 | 9.68E-04 |
| ENSG00000081189 | *MEF2C* | Day 7 | Survivor | -1.44 | 1.76E-03 |
| ENSG00000087245 | *MMP2* | Day 7 | Non-Survivor | 4.58 | 8.86E-05 |
| ENSG00000118113 | *MMP8* | Day 0 | Non-Survivor | 1.73 | 9.07E-03 |
| ENSG00000130830 | *MPP1* | Day 7 | Non-Survivor | 1.56 | 2.36E-03 |
| ENSG00000011028 | *MRC2* | Day 0 | Non-Survivor | 1.80 | 1.30E-03 |
| ENSG00000170873 | *MTSS1* | Day 7 | Survivor | -1.05 | 5.55E-03 |
| ENSG00000177034 | *MTX3* | Day 7 | Survivor | -1.11 | 7.25E-03 |
| ENSG00000119950 | *MXI1* | Day 0 | Non-Survivor | 1.85 | 2.27E-03 |
| ENSG00000134571 | *MYBPC3* | Day 7 | Non-Survivor | 1.71 | 2.83E-03 |
| ENSG00000078814 | *MYH7B* | Day 7 | Non-Survivor | 1.05 | 4.70E-03 |
| ENSG00000101605 | *MYOM1* | Day 7 | Non-Survivor | 1.30 | 1.84E-03 |
| ENSG00000279468 | *NA* | Day 0 | Non-Survivor | 1.36 | 9.65E-03 |
| ENSG00000102030 | *NAA10* | Day 7 | Non-Survivor | 1.22 | 9.68E-04 |
| ENSG00000186462 | *NAP1L2* | Day 7 | Survivor | -1.28 | 5.24E-03 |
| ENSG00000186310 | *NAP1L3* | Day 7 | Survivor | -1.51 | 3.26E-03 |
| ENSG00000123119 | *NECAB1* | Day 7 | Non-Survivor | 1.81 | 6.72E-03 |
| ENSG00000130202 | *NECTIN2* | Day 0 | Non-Survivor | 2.20 | 7.90E-03 |
| ENSG00000130202 | *NECTIN2* | Day 7 | Non-Survivor | 2.68 | 1.19E-03 |
| ENSG00000049759 | *NEDD4L* | Day 0 | Non-Survivor | 1.50 | 2.36E-03 |
| ENSG00000008441 | *NFIX* | Day 0 | Non-Survivor | 2.07 | 2.09E-04 |
| ENSG00000008441 | *NFIX* | Day 7 | Non-Survivor | 1.98 | 5.45E-03 |
| ENSG00000171786 | *NHLH1* | Day 7 | Non-Survivor | 1.44 | 6.58E-03 |
| ENSG00000183072 | *NKX2-5* | Day 0 | Non-Survivor | 2.56 | 4.75E-03 |
| ENSG00000167634 | *NLRP7* | Day 7 | Non-Survivor | 1.35 | 5.51E-03 |
| ENSG00000198929 | *NOS1AP* | Day 0 | Non-Survivor | 2.02 | 4.89E-03 |
| ENSG00000143257 | *NR1I3* | Day 7 | Non-Survivor | 1.35 | 5.24E-03 |
| ENSG00000151623 | *NR3C2* | Day 7 | Survivor | -1.19 | 4.70E-03 |
| ENSG00000074527 | *NTN4* | Day 7 | Survivor | -3.16 | 3.48E-05 |
| ENSG00000138315 | *OIT3* | Day 7 | Non-Survivor | 2.03 | 7.15E-04 |
| ENSG00000116774 | *OLFML3* | Day 7 | Non-Survivor | 2.03 | 4.53E-03 |
| ENSG00000203907 | *OOEP* | Day 7 | Non-Survivor | 2.22 | 9.65E-03 |
| ENSG00000116329 | *OPRD1* | Day 7 | Non-Survivor | 1.11 | 8.96E-03 |
| ENSG00000238243 | *OR2W3* | Day 0 | Non-Survivor | 2.79 | 1.35E-03 |
| ENSG00000184792 | *OSBP2* | Day 0 | Non-Survivor | 2.08 | 1.35E-03 |
| ENSG00000169918 | *OTUD7A* | Day 7 | Survivor | -1.44 | 4.07E-03 |
| ENSG00000238269 | *PAGE2B* | Day 0 | Non-Survivor | 2.65 | 8.90E-03 |
| ENSG00000129116 | *PALLD* | Day 7 | Survivor | -1.29 | 7.27E-03 |
| ENSG00000188582 | *PAQR9* | Day 0 | Non-Survivor | 3.08 | 1.98E-03 |
| ENSG00000148498 | *PARD3* | Day 7 | Non-Survivor | 1.22 | 3.51E-03 |
| ENSG00000173200 | *PARP15* | Day 7 | Survivor | -1.47 | 3.47E-03 |
| ENSG00000185630 | *PBX1* | Day 7 | Non-Survivor | 1.80 | 7.84E-03 |
| ENSG00000197461 | *PDGFA* | Day 7 | Non-Survivor | 1.44 | 1.54E-03 |
| ENSG00000004799 | *PDK4* | Day 7 | Survivor | -1.48 | 6.01E-03 |
| ENSG00000162366 | *PDZK1IP1* | Day 7 | Non-Survivor | 2.17 | 5.50E-03 |
| ENSG00000163737 | *PF4* | Day 7 | Non-Survivor | 1.48 | 7.11E-03 |
| ENSG00000163218 | *PGLYRP4* | Day 7 | Non-Survivor | 2.40 | 1.93E-03 |
| ENSG00000112137 | *PHACTR1* | Day 7 | Non-Survivor | 1.83 | 1.67E-04 |
| ENSG00000124102 | *PI3* | Day 7 | Non-Survivor | 2.10 | 4.70E-03 |
| ENSG00000137193 | *PIM1* | Day 7 | Non-Survivor | 1.35 | 9.19E-03 |
| ENSG00000087842 | *PIR* | Day 7 | Non-Survivor | 2.48 | 6.58E-03 |
| ENSG00000143627 | *PKLR* | Day 7 | Non-Survivor | 3.48 | 6.25E-03 |
| ENSG00000067225 | *PKM* | Day 7 | Non-Survivor | 1.46 | 1.84E-03 |
| ENSG00000179598 | *PLD6* | Day 7 | Non-Survivor | 1.06 | 2.27E-03 |
| ENSG00000100558 | *PLEK2* | Day 0 | Non-Survivor | 2.42 | 2.61E-03 |
| ENSG00000008323 | *PLEKHG6* | Day 7 | Non-Survivor | 1.58 | 3.18E-03 |
| ENSG00000032444 | *PNPLA6* | Day 7 | Non-Survivor | 1.07 | 9.68E-04 |
| ENSG00000155846 | *PPARGC1B* | Day 7 | Survivor | -1.13 | 2.62E-03 |
| ENSG00000163736 | *PPBP* | Day 7 | Non-Survivor | 1.41 | 4.57E-03 |
| ENSG00000125534 | *PPDPF* | Day 7 | Non-Survivor | 1.63 | 4.96E-03 |
| ENSG00000185686 | *PRAME* | Day 7 | Non-Survivor | 5.22 | 7.76E-03 |
| ENSG00000019485 | *PRDM11* | Day 7 | Survivor | -1.11 | 1.70E-03 |
| ENSG00000115592 | *PRKAG3* | Day 7 | Non-Survivor | 3.02 | 2.27E-03 |
| ENSG00000010438 | *PRSS3* | Day 7 | Non-Survivor | 3.23 | 3.69E-03 |
| ENSG00000206549 | *PRSS50* | Day 7 | Non-Survivor | 2.96 | 5.86E-03 |
| ENSG00000159335 | *PTMS* | Day 7 | Non-Survivor | 1.93 | 2.37E-03 |
| ENSG00000142949 | *PTPRF* | Day 7 | Non-Survivor | 1.82 | 6.72E-03 |
| ENSG00000068976 | *PYGM* | Day 7 | Non-Survivor | 1.62 | 3.85E-04 |
| ENSG00000139832 | *RAB20* | Day 7 | Non-Survivor | 1.69 | 8.71E-03 |
| ENSG00000167994 | *RAB3IL1* | Day 7 | Non-Survivor | 2.27 | 6.58E-03 |
| ENSG00000076864 | *RAP1GAP* | Day 0 | Non-Survivor | 2.94 | 4.95E-03 |
| ENSG00000076864 | *RAP1GAP* | Day 7 | Non-Survivor | 5.16 | 8.86E-05 |
| ENSG00000132819 | *RBM38* | Day 0 | Non-Survivor | 1.56 | 4.92E-03 |
| ENSG00000100918 | *REC8* | Day 7 | Non-Survivor | 1.05 | 8.23E-03 |
| ENSG00000132563 | *REEP2* | Day 7 | Non-Survivor | 2.49 | 1.19E-03 |
| ENSG00000102032 | *RENBP* | Day 7 | Non-Survivor | 1.07 | 2.52E-03 |
| ENSG00000112077 | *RHAG* | Day 0 | Non-Survivor | 3.68 | 1.80E-03 |
| ENSG00000112077 | *RHAG* | Day 7 | Non-Survivor | 3.90 | 9.68E-04 |
| ENSG00000005486 | *RHBDD2* | Day 7 | Non-Survivor | 1.10 | 5.50E-03 |
| ENSG00000158315 | *RHBDL2* | Day 7 | Non-Survivor | 2.36 | 5.12E-03 |
| ENSG00000188672 | *RHCE* | Day 0 | Non-Survivor | 2.83 | 6.22E-04 |
| ENSG00000188672 | *RHCE* | Day 7 | Non-Survivor | 3.51 | 2.60E-05 |
| ENSG00000104140 | *RHOV* | Day 7 | Non-Survivor | 4.62 | 2.88E-03 |
| ENSG00000188026 | *RILPL1* | Day 7 | Non-Survivor | 1.14 | 2.83E-03 |
| ENSG00000212864 | *RNF208* | Day 7 | Non-Survivor | 1.34 | 9.19E-03 |
| ENSG00000149489 | *ROM1* | Day 7 | Non-Survivor | 1.16 | 4.38E-03 |
| ENSG00000089169 | *RPH3A* | Day 7 | Non-Survivor | 2.89 | 5.45E-04 |
| ENSG00000125744 | *RTN2* | Day 7 | Non-Survivor | 1.78 | 2.75E-03 |
| ENSG00000108309 | *RUNDC3A* | Day 0 | Non-Survivor | 2.51 | 5.79E-04 |
| ENSG00000108309 | *RUNDC3A* | Day 7 | Non-Survivor | 2.89 | 2.52E-03 |
| ENSG00000198626 | *RYR2* | Day 7 | Non-Survivor | 2.59 | 6.10E-04 |
| ENSG00000196154 | *S100A4* | Day 7 | Non-Survivor | 1.28 | 9.53E-03 |
| ENSG00000167100 | *SAMD14* | Day 7 | Non-Survivor | 1.69 | 7.84E-03 |
| ENSG00000145284 | *SCD5* | Day 0 | Survivor | -1.56 | 8.01E-03 |
| ENSG00000149575 | *SCN2B* | Day 7 | Non-Survivor | 2.29 | 8.93E-04 |
| ENSG00000100012 | *SEC14L3* | Day 0 | Non-Survivor | 2.70 | 6.19E-03 |
| ENSG00000133488 | *SEC14L4* | Day 0 | Non-Survivor | 3.03 | 2.55E-03 |
| ENSG00000143416 | *SELENBP1* | Day 0 | Non-Survivor | 2.50 | 6.10E-03 |
| ENSG00000174175 | *SELP* | Day 7 | Non-Survivor | 1.69 | 7.89E-04 |
| ENSG00000138623 | *SEMA7A* | Day 7 | Non-Survivor | 1.58 | 7.96E-03 |
| ENSG00000214193 | *SH3D21* | Day 7 | Non-Survivor | 1.23 | 2.09E-05 |
| ENSG00000181788 | *SIAH2* | Day 0 | Non-Survivor | 1.35 | 9.07E-03 |
| ENSG00000144671 | *SLC22A14* | Day 7 | Non-Survivor | 1.77 | 1.84E-03 |
| ENSG00000182902 | *SLC25A18* | Day 7 | Non-Survivor | 1.59 | 3.69E-03 |
| ENSG00000225697 | *SLC26A6* | Day 7 | Non-Survivor | 1.70 | 5.45E-04 |
| ENSG00000117394 | *SLC2A1* | Day 0 | Non-Survivor | 1.54 | 4.27E-03 |
| ENSG00000117394 | *SLC2A1* | Day 7 | Non-Survivor | 1.93 | 5.45E-04 |
| ENSG00000168003 | *SLC3A2* | Day 7 | Non-Survivor | 2.05 | 9.68E-04 |
| ENSG00000158715 | *SLC45A3* | Day 7 | Non-Survivor | 1.87 | 2.97E-03 |
| ENSG00000004939 | *SLC4A1* | Day 0 | Non-Survivor | 2.62 | 2.61E-03 |
| ENSG00000115616 | *SLC9A2* | Day 7 | Non-Survivor | 1.92 | 9.65E-03 |
| ENSG00000065923 | *SLC9A7* | Day 7 | Survivor | -1.12 | 4.96E-03 |
| ENSG00000130768 | *SMPDL3B* | Day 7 | Non-Survivor | 1.68 | 1.70E-03 |
| ENSG00000145335 | *SNCA* | Day 0 | Non-Survivor | 2.10 | 2.61E-03 |
| ENSG00000095637 | *SORBS1* | Day 7 | Non-Survivor | 1.44 | 2.83E-03 |
| ENSG00000198142 | *SOWAHC* | Day 7 | Survivor | -1.17 | 6.01E-03 |
| ENSG00000124766 | *SOX4* | Day 7 | Survivor | -1.15 | 5.69E-03 |
| ENSG00000158792 | *SPATA2L* | Day 7 | Non-Survivor | 1.14 | 8.08E-03 |
| ENSG00000164056 | *SPRY1* | Day 7 | Survivor | -1.66 | 9.15E-03 |
| ENSG00000163554 | *SPTA1* | Day 0 | Non-Survivor | 3.32 | 3.08E-04 |
| ENSG00000163554 | *SPTA1* | Day 7 | Non-Survivor | 2.65 | 5.88E-03 |
| ENSG00000070182 | *SPTB* | Day 0 | Non-Survivor | 2.71 | 2.09E-04 |
| ENSG00000214530 | *STARD10* | Day 7 | Non-Survivor | 1.74 | 2.37E-03 |
| ENSG00000082146 | *STRADB* | Day 0 | Non-Survivor | 2.05 | 1.22E-03 |
| ENSG00000102003 | *SYP* | Day 7 | Non-Survivor | 1.13 | 5.89E-03 |
| ENSG00000162367 | *TAL1* | Day 7 | Non-Survivor | 1.95 | 8.72E-03 |
| ENSG00000168394 | *TAP1* | Day 7 | Non-Survivor | 1.37 | 6.18E-03 |
| ENSG00000173991 | *TCAP* | Day 7 | Non-Survivor | 1.33 | 1.67E-04 |
| ENSG00000106327 | *TFR2* | Day 0 | Non-Survivor | 1.52 | 8.38E-03 |
| ENSG00000106327 | *TFR2* | Day 7 | Non-Survivor | 1.79 | 7.25E-03 |
| ENSG00000042832 | *TG* | Day 7 | Non-Survivor | 1.55 | 4.70E-03 |
| ENSG00000140682 | *TGFB1I1* | Day 7 | Non-Survivor | 1.82 | 1.67E-04 |
| ENSG00000069702 | *TGFBR3* | Day 7 | Survivor | -1.38 | 4.70E-03 |
| ENSG00000156299 | *TIAM1* | Day 7 | Survivor | -1.05 | 8.18E-03 |
| ENSG00000145365 | *TIFA* | Day 7 | Non-Survivor | 1.42 | 1.84E-03 |
| ENSG00000133069 | *TMCC2* | Day 0 | Non-Survivor | 2.87 | 5.80E-04 |
| ENSG00000133069 | *TMCC2* | Day 7 | Non-Survivor | 3.02 | 1.76E-03 |
| ENSG00000205090 | *TMEM240* | Day 7 | Non-Survivor | 2.28 | 1.70E-03 |
| ENSG00000121900 | *TMEM54* | Day 7 | Non-Survivor | 3.77 | 1.54E-03 |
| ENSG00000137216 | *TMEM63B* | Day 7 | Non-Survivor | 1.46 | 1.54E-03 |
| ENSG00000174529 | *TMEM81* | Day 7 | Non-Survivor | 1.30 | 7.15E-04 |
| ENSG00000136842 | *TMOD1* | Day 0 | Non-Survivor | 2.56 | 6.22E-04 |
| ENSG00000178297 | *TMPRSS9* | Day 0 | Non-Survivor | 1.82 | 1.97E-03 |
| ENSG00000178297 | *TMPRSS9* | Day 7 | Non-Survivor | 2.02 | 1.99E-03 |
| ENSG00000105048 | *TNNT1* | Day 7 | Non-Survivor | 2.83 | 2.76E-03 |
| ENSG00000079308 | *TNS1* | Day 0 | Non-Survivor | 2.64 | 5.04E-04 |
| ENSG00000181029 | *TRAPPC5* | Day 7 | Non-Survivor | 1.39 | 2.97E-03 |
| ENSG00000161911 | *TREML1* | Day 7 | Non-Survivor | 1.99 | 7.89E-04 |
| ENSG00000162722 | *TRIM58* | Day 0 | Non-Survivor | 2.37 | 6.50E-04 |
| ENSG00000182704 | *TSKU* | Day 7 | Non-Survivor | 3.25 | 7.11E-03 |
| ENSG00000168785 | *TSPAN5* | Day 0 | Non-Survivor | 1.72 | 1.35E-03 |
| ENSG00000112212 | *TSPO2* | Day 0 | Non-Survivor | 3.00 | 1.13E-03 |
| ENSG00000112212 | *TSPO2* | Day 7 | Non-Survivor | 3.13 | 1.47E-03 |
| ENSG00000204815 | *TTC25* | Day 0 | Non-Survivor | 1.70 | 4.75E-03 |
| ENSG00000085831 | *TTC39A* | Day 7 | Non-Survivor | 1.81 | 8.03E-04 |
| ENSG00000127824 | *TUBA4A* | Day 7 | Non-Survivor | 1.11 | 7.19E-03 |
| ENSG00000188229 | *TUBB4B* | Day 7 | Non-Survivor | 1.04 | 2.27E-03 |
| ENSG00000136810 | *TXN* | Day 7 | Non-Survivor | 1.29 | 4.99E-03 |
| ENSG00000137831 | *UACA* | Day 7 | Survivor | -1.42 | 8.82E-03 |
| ENSG00000185262 | *UBALD2* | Day 7 | Non-Survivor | 1.36 | 8.87E-03 |
| ENSG00000170315 | *UBB* | Day 0 | Non-Survivor | 1.55 | 8.58E-04 |
| ENSG00000130725 | *UBE2M* | Day 7 | Non-Survivor | 1.01 | 1.72E-03 |
| ENSG00000100024 | *UPB1* | Day 7 | Non-Survivor | 1.89 | 5.45E-04 |
| ENSG00000112290 | *WASF1* | Day 7 | Non-Survivor | 1.12 | 2.74E-03 |
| ENSG00000185222 | *WBP5* | Day 7 | Non-Survivor | 1.42 | 9.84E-05 |
| ENSG00000127578 | *WFIKKN1* | Day 7 | Non-Survivor | 1.42 | 3.56E-03 |
| ENSG00000047597 | *XK* | Day 0 | Non-Survivor | 2.06 | 9.12E-04 |
| ENSG00000172967 | *XKR3* | Day 7 | Non-Survivor | 2.16 | 4.07E-03 |
| ENSG00000060138 | *YBX3* | Day 0 | Non-Survivor | 1.84 | 1.37E-03 |
| ENSG00000090238 | *YPEL3* | Day 7 | Non-Survivor | 1.19 | 6.60E-03 |
| ENSG00000166793 | *YPEL4* | Day 0 | Non-Survivor | 1.96 | 4.75E-03 |
| ENSG00000166793 | *YPEL4* | Day 7 | Non-Survivor | 3.08 | 6.88E-04 |
| ENSG00000205189 | *ZBTB10* | Day 7 | Survivor | -1.05 | 1.92E-03 |
| ENSG00000184307 | *ZDHHC23* | Day 7 | Survivor | -1.32 | 5.25E-03 |
| ENSG00000220201 | *ZGLP1* | Day 7 | Non-Survivor | 1.19 | 6.37E-03 |
| ENSG00000179909 | *ZNF154* | Day 7 | Survivor | -1.60 | 4.10E-03 |
| ENSG00000138311 | *ZNF365* | Day 7 | Survivor | -1.87 | 1.81E-03 |
| ENSG00000074657 | *ZNF532* | Day 7 | Survivor | -1.49 | 1.84E-03 |
| ENSG00000204519 | *ZNF551* | Day 7 | Survivor | -1.05 | 2.76E-03 |
| ENSG00000197497 | *ZNF665* | Day 7 | Survivor | -1.57 | 4.03E-03 |
| ENSG00000197928 | *ZNF677* | Day 7 | Survivor | -1.64 | 1.19E-03 |
